# Genomic insight into balancing high yield, good quality, and blast resistance of *japonica* rice

**DOI:** 10.1186/s13059-021-02488-8

**Published:** 2021-10-06

**Authors:** Ning Xiao, Cunhong Pan, Yuhong Li, Yunyu Wu, Yue Cai, Yue Lu, Ruyi Wang, Ling Yu, Wei Shi, Houxiang Kang, Zhaobing Zhu, Niansheng Huang, Xiaoxiang Zhang, Zichun Chen, Jianju Liu, Zefeng Yang, Yuese Ning, Aihong Li

**Affiliations:** 1 Institute of Agricultural Sciences for Lixiahe Region in Jiangsu, Yangzhou, 225009 China; 2 State Key Laboratory for Biology of Plant Diseases and Insect Pests, Institute of Plant Protection, Chinese Academy of Agricultural Sciences, Beijing, 100193 China; 3 Key Laboratory of Plant Functional Genomics, Ministry of Education, Jiangsu Key Laboratory of Crop Genomics and Molecular Breeding, Agricultural College of Yangzhou University, Yangzhou, 225009 China; 4 Jiangsu Collaborative Innovation Center for Modern Crop Production, Nanjing, 210095 China

**Keywords:** Rice, High yield, Excellent taste quality, Disease resistance, Genomic landscape, Molecular design breeding

## Abstract

**Background:**

Balancing the yield, quality and resistance to disease is a daunting challenge in crop breeding due to the negative relationship among these traits. Large-scale genomic landscape analysis of germplasm resources is considered to be an efficient approach to dissect the genetic basis of the complex traits. Central China is one of the main regions where the *japonica* rice is produced. However, dozens of high-yield rice varieties in this region still exist with low quality or susceptibility to blast disease, severely limiting their application in rice production.

**Results:**

Here, we re-sequence 200 *japonica* rice varieties grown in central China over the past 30 years and analyze the genetic structure of these cultivars using 2.4 million polymorphic SNP markers. Genome-wide association mapping and selection scans indicate that strong selection for high-yield and taste quality associated with low-amylose content may have led to the loss of resistance to the rice blast fungus *Magnaporthe oryzae*. By extensive bioinformatic analyses of yield components, resistance to rice blast, and taste quality, we identify several superior alleles for these traits in the population. Based on this information, we successfully introduce excellent taste quality and blast-resistant alleles into the background of two high-yield cultivars and develop two elite lines, XY99 and JXY1, with excellent taste, high yield, and broad-spectrum of blast resistance.

**Conclusions:**

This is the first large-scale genomic landscape analysis of *japonica* rice varieties grown in central China and we demonstrate a balancing of multiple agronomic traits by genomic-based strategy.

**Supplementary Information:**

The online version contains supplementary material available at 10.1186/s13059-021-02488-8.

## Background

Rice (*Oryza sativa* L.), one of the most important staple crops, feeds more than half of the population in the world. Although rice yield has undergone enormous leaps through semi-dwarfism and genetic heterosis in the last 60 years, rice production now faces several new challenges, including continuing population growth [1], a strong emphasis on cooking and taste quality with modern consumption structure shift [2], and increasing occurrence and new emergence of severe diseases owing to global climate change [3, 4]. Cultivation and application of green super rice with high yield, good quality and disease resistance is an irresistible trend [5].

Resource reallocation and genetic linkage mean that growth and immunity in plants are usually intertwined and are antagonistic [6]. Therefore, balancing yield, quality, and resistance to disease is a challenge in plant breeding. For rice, yield is a complex trait controlled by three main quantitative traits: panicle numbers, grain numbers per panicle, and grain weight, which are regulated by a large number of genes and some of the genes are highly pleiotropic [7–9]. Thus, optimal yield often comes with other unintended phenotypic effects [5]. Amylose content (AC), as the most important characteristic affecting taste quality, has been identified to be affected by multiple *Wx* alleles; however, the beneficial alleles rarely occur in most of varieties [2, 10]. Rice blast, caused by the fungal pathogen *Magnaporthe oryzae*, is the most devastating fungal disease to rice production worldwide [11]. Recent emerging studies showed that the nucleotide-binding leucine-rich repeat receptor *Pigm* confers blast resistance with yield balance through epigenetic regulation [12] and a natural allele of a C2H2-type transcription factor *Bsr-d1* possesses enhanced blast resistance with no observable penalty in plant growth or yield by inhibition of H_2_O_2_ degradation [13]. It is undeniable that hundreds of valuable genes relate to yield, quality, and blast resistance have been identified in rice [14–18] and the delicate balance of yield and immunity have been significantly developed, but the widespread phenomena of “high yield without disease resistance” or “good quality without high yield” is widely observed in crop breeding science [19, 20]. So far, few studies have demonstrated the balancing immunity and good quality. Thus, it is difficult to generate novel elite varieties with high yield, good taste quality, and enhanced resistance through traditional approach. Therefore, illustrating the superior genes that could generate novel elite varieties with high yield, good taste quality, and enhanced resistance, and developing the key technical strategy to achieve multi-gene aggregation are big challenges in breeding science.

Since the release of the first rice genome in 2005 [21], generation of multiple genome assemblies per crop species is now a reality [22, 23]. It has provided a golden opportunity that genome-wide dissection of the regulatory networks underlying the hub genes that are responsible for yield, taste quality, and blast resistance. Gene pyramiding molecular design is suggested to be an efficient breeding strategy in improving multiple traits [24]. However, it is very difficult to pyramid multiple target traits in molecular design breeding because of the uncertainty of genetic structure for existing breeding population and the lack of high-throughput genotyping for genomic selection. Therefore, illustrating superior alleles of yield, taste quality, and blast resistance and drawing whole-genome linkage in a particular breeding population will benefit to know how to balance multiple agronomic traits based on genomic-based strategy and perform breeding in a rapid, high-throughput, and precise manner.

The area of *japonica* rice grown in central China, which is one of the main regions producing *japonica* rice worldwide, exceeds 2 million hectares every year, and in the past 30 years, the yield of several rice varieties in this region has surpassed the yield of hybrid *indica* rice [25]. However, some *japonica* varieties still exist with low quality or susceptibility to blast disease, severely limiting their application in rice production. In this study, 200 *japonica* rice varieties grown in central China over the last 30 years were collected and re-sequenced to illustrate the genetic improvement laws relate to yield, quality, and blast resistance. Based on genome-wide association studies (GWAS) and selection scan results, we observed a key genomic linkage drag that caused excellent taste quality and high yield negatively correlated with rice blast resistance in the present breeding population. To overcome this dilemma, excellent taste quality or blast-resistant alleles have been introduced into the high-yield cultivars. Finally, we successfully bred two elite lines with a yield, quality, and blast resistance balance. This study develops a novel molecular design breeding strategy and closes the gaps between genomic studies and crop breeding.

## Results

### Genetic selection laws for yield and quality

To analyze the genomic landscape of *japonica* rice varieties in central China, we re-sequenced 200 *japonica* rice varieties bred from 1989 to 2017 in this region (Additional file 1: Fig. S1a); a *japonica* rice variety from Japan (*Nipponbare*) was used as a sequencing reference. A total of 2,410,743 single-nucleotide polymorphism (SNP) markers covering the whole genome (approximately 15× coverage) were obtained (Additional file 1: Fig. S1b). According to the significant decreasing trend of cross-validation error when the *K* value is nine, the sequenced varieties were distributed into nine groups (Additional file 1: Fig. S1c). Remarkably, the grouping of varieties was closely associated with the breeding area. For example, the varieties collected in Jiangsu province were distributed largely in groups 3, 4, and 5, whereas the varieties collected in the Shanghai area were individually assigned to group 7 (Additional file 1: Fig. S1d). These observations indicate significant diversity in the genomic landscape and genetic relationships among the varieties collected from different regions.

**Fig. 1:**
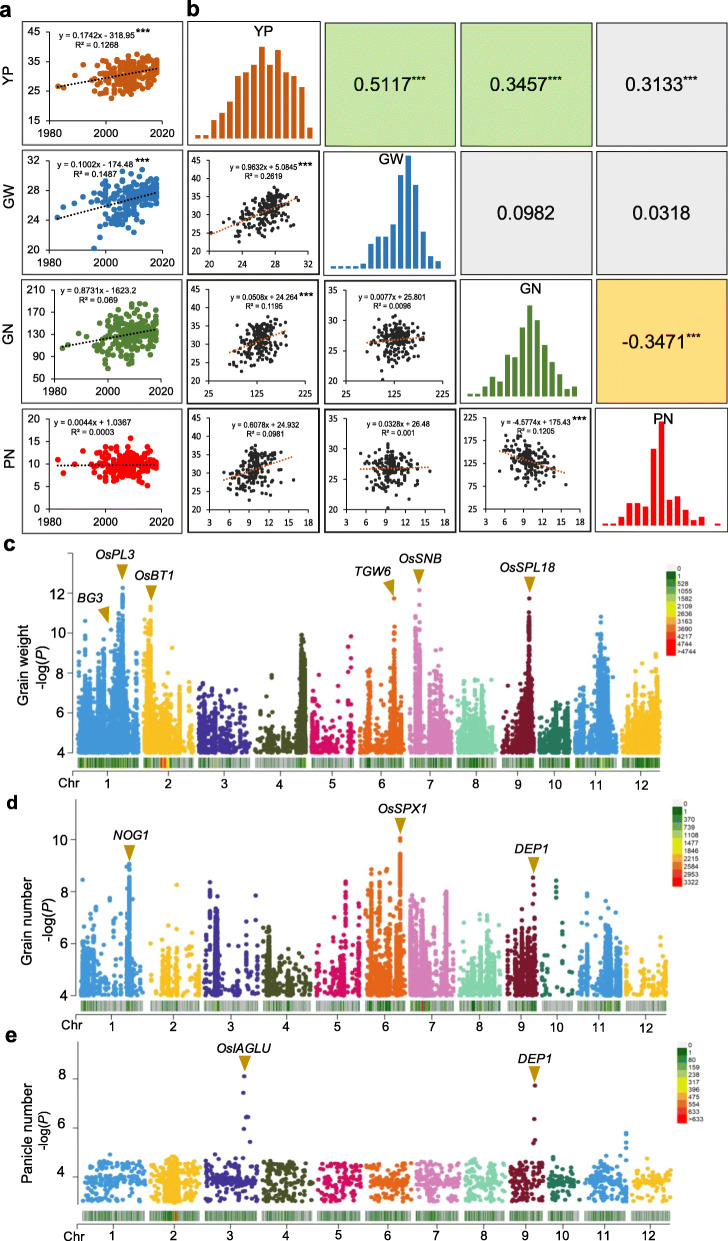
Genome-wide association studies of genes related to yield traits. **a** The change of yield traits of the sequenced 200 *japonica* rice varieties. *** indicates a significant correlation at *P* < 0.001, ** indicates a significant correlation at *P* < 0.01. **b** Correlation and Gaussian distribution of yield, grain weight, grain number, and panicle traits; the diagonals represent the distribution of different yield traits. *** indicates a significant correlation (*P* < 0.001). **c–e** Manhattan plots of genome-wide association studies (GWASs) of grain weight, grain number, and panicle number using 200 *japonica* rice varieties; minor allele frequency (MAF) > 0.05. The threshold − log(*P*) value of the GWAS result was 8.3. The heatmaps in **c–e** show the density of SNP markers (− log(*P*) value > 4) in 100 kb chromosome intervals. YP, yield per plant (g); GW, 1000-grain weight (g); PN, panicle number; GN, grain number

To investigate the genetic impact of selection during modern rice breeding and to identify the key genes contributing to adaptation to increased yield, four key agronomic traits, including yield per plant (YP), 1000-grain weight (GW), grain number per plant (GN), and panicle per plant (PN), were identified in four environments (Additional file 2: Table S1). ANOVA results revealed that the rice varieties (genotypes) showed statistically significant variance for all four traits in the four environments (Additional file 2: Table S2 and S3), suggesting that the selected rice varieties hold diverse characteristics for association analysis. Therefore, the above four trait values in the four environments were fitted using a linear mixed model in *lme4* R package to obtain a best linear unbiased predictor (BLUP) value. Based on these predicted values, we noticed that the yield, GW and GN increased over the past 30 years, while PN did not significantly change (Fig. 1a). The yield correlated significantly and positively with the 1000-grain weight (*r* = 0.5117; *p*<0.001), the number of grains per panicle (*r* = 0.3457; *P* < 0.001), and the panicle number (*r* = 0.3133; *P* < 0.001; Fig. 1b), indicating that the increase of grain weight and grain number per panicle are two main factors for the yield increase of *japonica* rice in central China. Yield is a complex quantitative trait controlled by multiple major and minor genes; to understand the critical yield-related genes in the 200 sequenced *japonica* rice varieties, we used GWAS with a mixed linear model (MLM) to further detect the gene loci related to GW, GN, and PN. The results revealed that the MLM model was appropriate to analyze these traits (Additional file 1: Fig S2), and a total of 20 significant loci (threshold − log(*P*) value > 8.3) were identified as being associated with these yield-related traits (Fig. 1c-d, Additional file 2: Table S4). Based on the significant SNP positions and the LD decay blocks, eleven loci were identified as previously reported quantitative trait loci for which the functional genes have been cloned, including six GW-related genes (*BG3* [26, 27], *OsPL3* [28], *OsBT1* [29], *TGW6* [30], *OsSNB* [31], and O*sSPL18* [32]) (Additional file 1: Fig. S3a); three GN-related genes (*NOG1* [33], *OsSPX1* [34], and *DEP1* [14, 35]) (Additional file 1: Fig. S3b), and two PN-related genes (*OsIAGLU* [36] and *DEP1*) (Additional file 1: Fig. S3c). In addition, we found that the varieties with multiple favorable alleles, including GW and GN, had a higher yield potential (Additional file 1: Fig. S4).

**Fig. 2:**
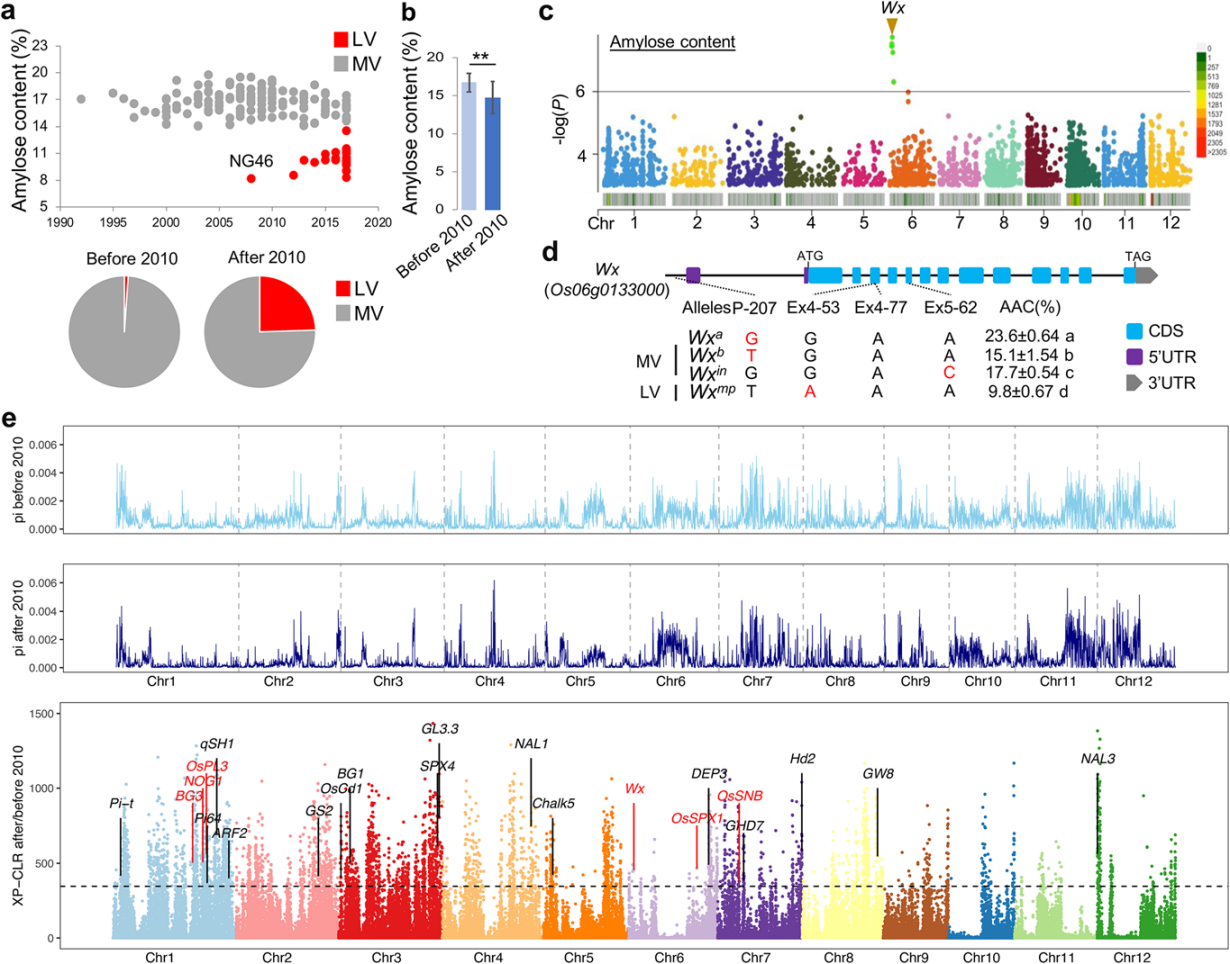
Identification of *Wx* allelic type for superior taste quality and genomic selection signals. **a** Amylose content of the sequenced 200 *japonica* rice varieties. NG46 with *Wx*^*mp*^ was the first LV released in 2008. LV, low-amylose (8–12%) variety; MV, medium-amylose (13–18%) variety. **b** The average amylose content showed a significant downward trend after 2010; ** indicates independent-samples *t*-test with *P* < 0.001; **c** Genetic loci associated with rice amylose content were identified by a genome-wide association study (GWAS) using 200 *japonica* rice varieties. Heatmap showing the density of SNP markers (− log(*P*) value > 4) in 100 kb chromosome intervals. **d** Key SNP variations that could distinguish four allelic variants (*Wx*^*a*^, *Wx*^*b*^, *Wx*^*in*^, and *Wx*^*mp*^) of the *Wx* locus. **e** Genome-wide genetic diversity (*π*) and cross-population composite likelihood ratio (XP-CLR) of varieties bred before 2010 and after 2010. Genes or QTLs related to grain weight, grain number, panicle number, and amylose content that were located in selection sweeps are indicated, and the loci identified in GWAS in this study are marked in red

Amylose content (AC) is one of the essential traits that influence rice taste quality, and low-amylose varieties usually have better-taste quality [10]. Nangeng 46 (NG46) was the first good-tasting *japonica* rice variety with low AC bred in 2008 from central China, and then a series of low AC varieties were derived from it (Fig. 2a), which directly lead to a significant decline in the AC of the selected varieties after 2010 (Fig. 2b and Additional file 1: Fig.S3d). Using the GWAS strategy described above, we found that an important gene, *Wx*, located at 1.67 Mb on chromosome 6 (Fig. 2c), regulates the AC. Further analysis showed that 22 low-amylose varieties (LV) with 8–12% AC mainly carried the *Wx*^*mp*^ genotype, whereas 172 medium-amylose varieties (MV) with 14–18% AC mainly carried the *Wx*^*b*^ genotype, and the other 3 MV varieties with 18–20% AC mainly carried the *Wx*^*in*^ genotype (Fig. 2d).

To further reveal whether these GWAS-mapped genes were artificially selected during the breeding process, we performed a cross-population composite likelihood ratio (XP-CLR) to scan the selective sweep regions at the genome-wide level. The results revealed that 655 regions were enhanced through artificial selection (Additional file 2: Table S5). Most of the genes related to grain weight, grain number per panicle, and taste quality identified by GWAS in the present study were included in these selective regions, including GW-related genes *BG3*, *OsPL3*, and *OsSNB*; GN-related genes *NOG1* and *OsSPX1*; and the amylose content-related gene *Wx* (Fig. 2e). Furthermore, the ratios of excellent alleles *NOG1*^*A*^, *OsSPX1*^*T*^, and *OsPL3*^*G*^ showed a significant increasing trend in the sub-population after 2010 (Additional file 1: Fig. S5a-b). It should be noted that yield improvement involves multiple genes, and the identified major genes have played an essential role in increasing the yield level and developing excellent taste characteristics in the past 30 years.

### *Piz* locus plays an important role in rice blast resistance improvement

**Fig. 3:**
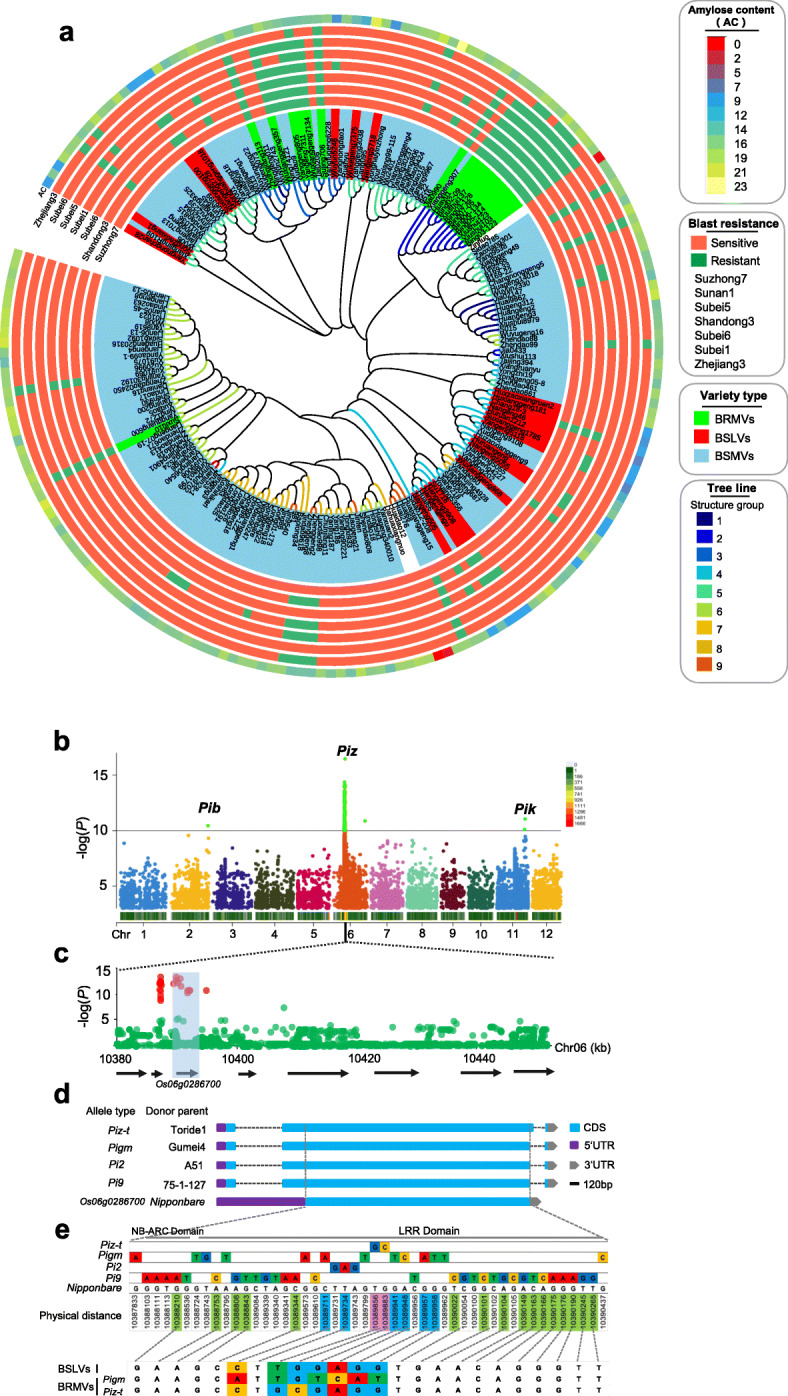
Identification of key alleles of the *Piz* locus contributing to blast resistance. **a** Neighbor-joining phylogenetic tree of the 200 *japonica* rice varieties with different blast resistance phenotypes and amylose content; the green blocks represent rice varieties that showed resistance to blast isolates. AC, amylose content; BSLVs, blast-sensitive and low-amylose (8–12%) varieties; BRMVs, blast-resistant and medium-amylose (13–18%) varieties. BSMVs, blast-sensitive, and medium-amylose (13–18%) varieties. **b** Blast-resistant associated loci were identified through a genome-wide association study (GWAS) using 200 *japonica* rice varieties, and single-nucleotide polymorphisms (SNPs) were filtered using a minor allele frequency (MAF) threshold of < 0.05. **c** Physical location of SNPs related to the *Piz* locus. **d** Comparing the coding frame homologous sequences among *Pi2*, *Pi9*, *Piz-t*, and *Pigm* from the donor parents A51, 75-1-127, Toride1, and Gumei4, respectively. **e** Identification of specific SNPs of *Pi2*, *Pi9*, *Piz-t*, and *Pigm* multiple alleles. The positions of the nucleotide-binding NB-ARC domain and the leucine-rich LRR domain are indicated

Rice blast, caused by the fungus *M. oryzae*, is arguably the most devastating fungal disease in rice. In recent years, more than 50% of the *japonica* rice-growing regions of central China have experienced a continuous outbreak of rice blast disease, presenting a massive threat to rice production [37]. To assess the blast resistance of the above 200 *japonica* rice varieties, we sequenced 35 isolates of *M. oryzae* collected from the main *japonica* rice-growing areas in the central region, including Jiangsu (including northern, central, and southern Jiangsu, named Subei, Suzhong, and Sunan), Zhejiang, and Shandong provinces, which are the main *japonica* rice-growing areas in the central region (Additional file 1: Fig. S6a). A total of 85,335 SNPs with a minor allele frequency (MAF) > 0.05 were obtained (30× coverage), using the *M. oryzae* isolate 70-15 genome as a reference (Additional file 1: Fig. S6b). Based on these SNPs, the 35 isolates were classified into five groups, and each group was closely related to the geographical location of strain collection (Additional file 1: Fig. S6c). Based on the genotype grouping and haplotype characteristics of the virulence genes of the above isolates (Additional file 1: Fig. S7a-b), seven representative isolates were selected to assess the pathogenesis using monogenic rice lines with a blast-sensitive variety LTH (Lijiangxintuanheigu) background. The results showed a one-to-one correspondence between the resistance genes carried by the monogenic rice lines and the avirulent genes in the *M. oryzae* isolates (Additional file 1: Fig. S6d). We then used the above seven representative isolates of *M. oryzae* to inoculate the 200 rice varieties, and found that only seven varieties were resistant to all seven isolates, while 150 of the 200 varieties were susceptible to all seven isolates. By combining the previously identified AC phenotype, all varieties could be classified into three types: (1) 18 varieties with 14–18% AC showing resistance to more than six isolates were classified to the blast-resistant, medium-amylose varieties (BRMVs); (2) 22 varieties with 8–12% AC showing susceptibility to more than three isolates classified to the blast-sensitive, low-amylose varieties (BSLVs); (3) 157 varieties with 14–18% AC showing susceptibility to more than three isolates classified to the blast-sensitive, medium-amylose varieties (BSMVs) (Fig. 3a).

We further investigated the blast-resistant genes in BRMVs using GWAS analysis and discovered that three dominant genetic loci (*Pib, Piz*, and *Pik*) were associated with rice blast resistance on chromosomes 2, 6, and 11, respectively (Fig. 3b). Among them, *Piz* was the most significant resistance locus, and most of the significant SNPs (*P* < 10e^−10^) were distributed near the nucleotide-binding site–leucine-rich repeat (NBS-LRR) resistance gene (*Os06g0286700*) in the *Nipponbare* genome (Fig. 3c). Previously reported broad-spectrum resistance (BSR) genes were identified at the *Piz* locus, including *Pi2* [38], *Pi9* [39], *Piz-t* [40], and *Pigm* [12]; therefore, we amplified the coding sequences of these four BSR genes from the donor parents (A51, 75-1-127, Toride1, and Gumei4), and then compared them with the *Os06g0286700* coding sequence in *Nipponbare*. A total of 48 specific SNPs were identified that could distinguish multiple resistance allele genotypes (Fig. 3d). Among them, five SNPs were located in the NB-ARC domain, while 39 SNPs were located in the LRR domain. Finally, we identified two key SNPs, Chr6:10389856 and Chr6:10389883. The blast resistance allele genotypes in the *Piz-t* gene were “G” and “C,” but the susceptible genotypes and *Pi2*, *Pi9*, and *Pigm* were “T” and “G” (Fig. 3e). Additionally, we obtained three SNPs specific to *Pigm* and their resistance allele genotypes in Chr6:10389711, Chr6:10389941, and Chr6:10389948, which were “A,” “T,” and “C” respectively. The allelic genotypes analysis at the *Piz* locus of the 18 BRMVs showed that, besides the intermediate material “Yanggeng7311” carries *Pigm* gene, the other 17 *japonica* rice varieties from Jiaxing in Zhejiang province had the allelic genotype *Piz-t*, which is the primary blast resistance gene used in *japonica* rice varieties in central China.

### Genomic linkage drag caused bred failure for varieties with excellent taste and blast resistance

**Fig. 4:**
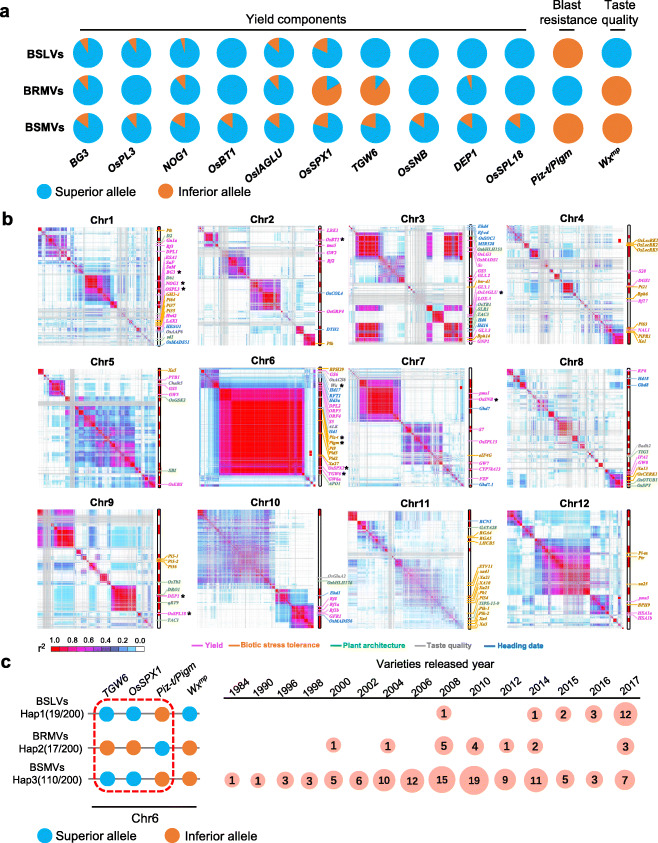
Rice genomic distribution and linkage drag for yield, taste quality, and blast resistance associated genes. **a** Allelic frequency of superior alleles related to yield, taste quality, and blast resistance in BSLVs, BRMVs, and BSMVs. **b** Genomic linkage disequilibrium blocks drawn through genome-wide SNPs with a 15-kb sliding window size advantage, and the genomic locations of the 158 genes related to yield, rice quality, and resistance are illustrated along the 12 rice chromosomes. The functional category of each gene is indicated by colored lines pointing at the gene labels. Each chromosome in the segments represents a genetic drag with *R*^2^ > 0.95. Linked genes on chromosomes marked with red dashed boxes. **c** Schematic diagram of the genetic linkage among yield, good taste, and blast resistance genes on chromosome 6. Hap1 represents a high yield and excellent taste linkage type, mainly distributed in the BSLV. Hap2 represents low-yield and blast-resistant linkage types, mainly distributed in BRMV, and Hap3 represents the high-yield and blast susceptible linkage type, mainly distributed in the BSRM. The distribution of the above three linkage types in varieties in different breeding years is indicated by light red circles with various numbers. Linked genes are denoted by red dashed boxes. BSLVs; blast-sensitive and low-amylose (8–12%) varieties; BRMVs; blast-resistant and medium-amylose (13–18%) varieties

The distribution of superior alleles, including yield, excellent taste, and blast resistance genes identified by GWAS among BSLV, BRMVs, and BSMVs, revealed that BSLVs had pyramided more superior alleles related to yield and excellent taste than those in the BRMVs and BSMVs, but the major blast-resistant gene *Piz-t/Pigm* was lost (Fig. 4a). Although BRMVs had major blast-resistant genes, the frequency of superior alleles of yield was lower than that of BSLVs. For example, the ratios of the superior alleles of *OsSPX1* and *TGW6* in *BRMVs* were only 16.7% and 11.1% (Fig. 4a), respectively. The most likely explanation for the unbalanced distribution of superior alleles among different variations might be associated with genetic linkage drag. To dissect whole-genomic drag hots based on the 200 sequenced cultivars, we used genome-wide SNP markers with an average interval of 10 kb to construct all linkage disequilibrium (LD) blocks for 12 chromosomes (Fig. 4b). Moreover, a total of 158 known genes, which are reported to be related to the yield, disease resistance, taste quality, and heading day traits, were plotted on all chromosomes (Fig. 4b and Additional file 2: Table S6). Among them, 13 genes were detected using GWAS in this study. We also noticed that the distribution of the genomic drags was quite uneven in rice chromosomes. Only a few drags with block size < 3 Mb were found on chromosomes 4, 8, and 11, while some larger blocks (size > 6 Mb) were on chromosomes 1, 3, 6, and 9. Among them, the longest block was found in chromosome 6, ranging from 5.3 to 29.4 Mb (Fig. 4b). Multiple genes with important breeding values were located in this block, including the yield-related genes *TGW6*, *SPX1*, and *GW6a*, the blast-resistant gene *Piz-t/Pigm*, the paste-temperature gene *ALK*, and the heading-day gene *Hd1*. In addition, some other functional genes were identified in the same block. For example, seven genes in chromosome 3, including three yield-related genes, *GL3.2*, *OsIAGLU*, and *LOX-3*, two heading-day genes, *Hd6* and *Hd16*, and the plant-type genes, *OsTB1*, *SLR1*, and *TAC3*, were found in the same block.

Furthermore, an important type of linkage drag (Hap2) was observed (Fig. 4c), in most BRMVs, the main alleles in *OsSPX1* and *TGW6* were typically inferior; however, *Piz-t/Pigm* was present as a superior allele. In other words, Hap2 was the respective low-yield and blast-resistant deleterious linkage type, which could cause opposite genetic phases during the breeding process (Additional file 1: Fig. S8a). When breeders intensified the artificial selection of high-yield traits, the plants carrying the inferior alleles of yield could be discarded, which may cause the superior major blast-resistant alleles to be removed precipitously in the breeding population (Additional file 1: Fig S8b). This hypothesis explained why the number of high-yield, excellent-tasting BSLV varieties bred in the past 10 years was increasing rapidly (Fig. 4c), while the superior major resistance gene *Piz-t/Pigm* was still not introduced at the *Piz* locus (Additional file 1: Fig. S9a). BSLVs have always maintained the inherent genome structure of high-yield and blast susceptible linkage types (Additional file 1: Fig. S9b). These results suggest that genetic drag is a common obstacle in rice breeding, and many adjacent genes tend to co-occur within the same block. During the breeding process, the introgression of superior alleles at some genes also introduced inferior alleles at linked loci during the breeding process due to high linkage. Hence, breaking these linkages, and pyramiding superior genes for high yield, excellent taste, and resistance is a key technical bottleneck that needs to be considered in molecular design breeding.

### Precise design of elite lines with high yield, excellent taste, and blast resistance

**Fig. 5:**
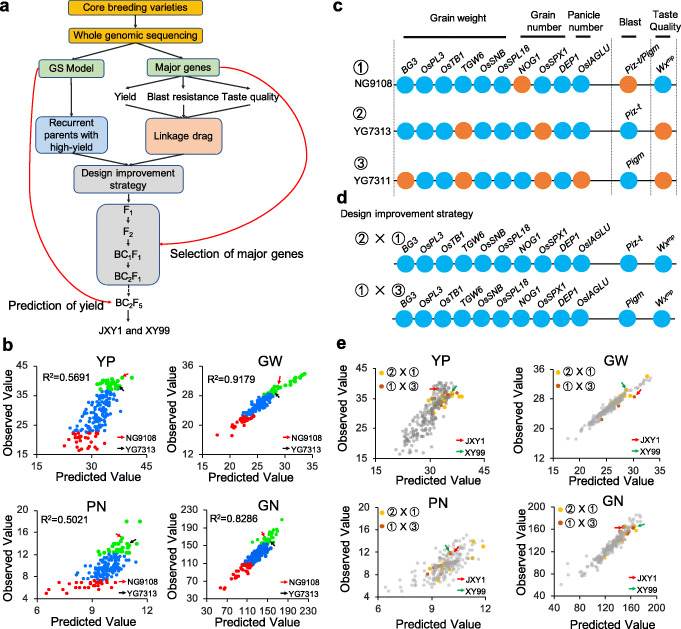
Molecular design strategy for pyramiding of superior genes related to yield, taste quality, and blast resistance. **a** Molecular design strategy for breeding novel *japonica* varieties with high yield, excellent taste quality, and blast resistance. **b** The accuracy of observed and predicted yield traits by rrBLUP model of 200 *japonica* rice varieties. YP, yield per plant (g); GW, 1000-grain weight (g); PN, panicle number per plant; GN, grain number per panicle. Based on the genomic selection model, YG7313 and NG9108 were selected as high-yield core parents. The green dots, blue dots, and red dots indicate the top 40, bottom 40, and other random varieties for YP, GW, GN, and PN, respectively. **c** Superior genes for yield, blast resistance, and excellent taste quality distributed in NG9108, YG7311, and YG7313. **d** Two improved strategies, ② × ①, respect the strategy of precise design of novel lines with high yield and excellent taste on the background of blast resistance through YG7311 as a recurrent parent. ① × ③ respects the strategy of new blast-resistant lines’ precise design on the background of high yield and excellent taste quality using NG9108 as a recurrent parent. **e** rrBLUP model was used to predict the yield, grain weight, grain number, and panicle number of recombination lines. Green arrows represent the elite line selected from the recombination lines with pyramiding of high yield, blast resistance, and excellent taste quality, named as “JXY1.” Red arrows represent the elite line selected from recombination lines with high yield, blast resistance, and excellent taste quality, named “XY99.” YP, yield per plant (g); GW, 1000-grain weight (g); PN, panicle number per plant; GN, grain number per panicle

Based on the above results on the GWAS identified major superior alleles for yield, taste quality, and blast resistance and whole-genomic linkage information, we proposed practical and constructive molecular design improvement strategies to breed novel *japonica* elite lines with high yield, excellent taste, and blast resistance (Fig. 5a), which include the following: (1) construction of a genomic selection (GS) model to pick core parents and elite recombination lines; (2) outlining a rational molecular improvement program; and (3) breaking linkage drag and breeding novel lines.

Yield is the basis of variety improvement; therefore, it is vital to select core parents with high yield potential. First, high-density SNP markers covering the whole genome were used to construct the yield GS prediction model. We used the 2,410,743 SNPs obtained above to construct a yield-prediction model based on the rrBLUP method to understand the selection of yield-related genes and found that the above models could predict traits such as yield, grain weight, grain number per panicle, and panicle number with high predictive power, with *R*^2^ values of 0.5691, 0.9179, 0.8286, and 0.5021, respectively (Fig. 5b). According to the above-predicted values and observed values in the field, two *japonica* rice varieties, YG7313 and NG9108, had high consistency between the predicted and observed values of yield-related parameters (Fig. 5b). Additionally, both had accumulated multiple alleles for grain weight and grain number per panicle (Fig. 5c). However, YG7313 lacked the excellent edible trait gene *Wx*^*mp*^, while NG9108 lacked the rice blast-resistant major gene, *Piz-t/Pigm*. Hence, both varieties have apparent drawbacks for rice production.

**Fig. 6:**
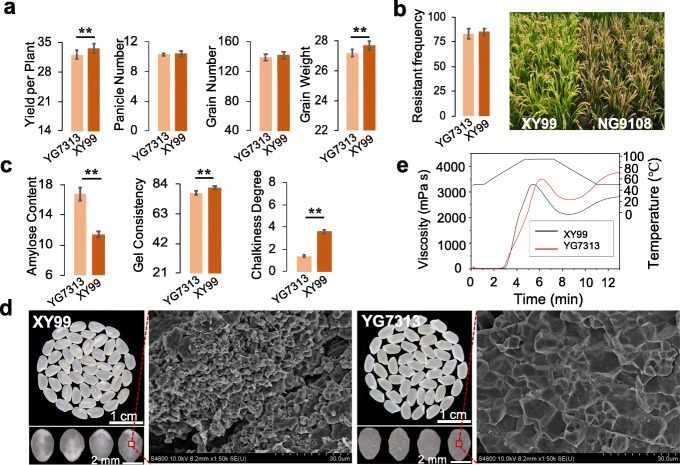
Excellent taste quality improvement in XY99. **a** Agronomic traits of XY99 and its recurrent parent YG7313. **b** Blast resistance of XY99, YG7313, and NG9108. **c** Significant differences were observed between XY99 carrying *Wx*^*mp*^ and its recurrent parent YG7313 in terms of amylose content, rice gel consistency, and chalkiness; ** *P* < 0.001 by Student’s *t* test. **d** Seed phenotype and starch granule morphology in XY99 and YG7313 were markedly different. **e** Rapid visco analyzer (RVA) results showing the pasting properties of milled rice in XY99 and YG7313

We designed two improved strategies based on the unbalanced appearances (Fig. 5d). The first strategy is the precise design of novel lines by introducing excellent edible trait gene in the background of high yield and blast resistance varieties. We chose YG7313 as the recurrent parent, and NG9108 as the donor parent of *Wx*^*mp*^, respectively. A large-scale F_2_ segregated population consisting of 5312 plants was used to break the linkage drag (Hap2), and finally we acquired twenty-four F_2_ plants with superior genes related to grain weight (*BG3*, *OsPL3*, *TGW6*, *OsSNB*, and *OsSPL18*), grain number (*NOG1*, *OsSPX1*, and *DEP1*), blast resistance (*Piz-t*), and excellent taste (*Wx*^*mp*^). Then, in each backcross and selfing generation, we strengthened the selection of the above superior gene. We then selected twenty BC_2_F_5_ generation lines for predicting the yield potential by constructing the rrBLUP prediction model. Finally, we selected one elite line (XY99) with high consistency between the predicted yield value and the observed yield value (Fig. 5e). XY99 retained the superior genes for grain weight, the grain number per panicle, effective panicle number, blast resistance from recurrent parent YG7313, and taste quality from the donor parent NG9108, respectively. In addition, we systematically analyzed the agronomic traits of XY99 and found that XY99 showed significantly higher grain weight and yield traits than recurrent parents because of the introduction of *TGW6* and *OsSPX1* from NG9108 (Fig. 6a). At the same time, XY99 maintained higher blast resistance frequency similar with YG7313 compared with the susceptible variety NG9108 (Fig. 6b). Remarkably, after introducing *Wx*^*mp*^, the AC of XY99 decreased to 11.4%, while the gel consistency increased to 81.2 mm (Fig. 6c). Compared to recurrent parent YG7313, the opaque endosperm of XY99 comprised small, round, loose composite starch granules (Fig. 6d). Furthermore, XY99 showed higher edible quality with lower viscosity (Fig. 6e), higher disintegration, and lower retrogradation (Additional file 2: Table S7).

**Fig. 7:**
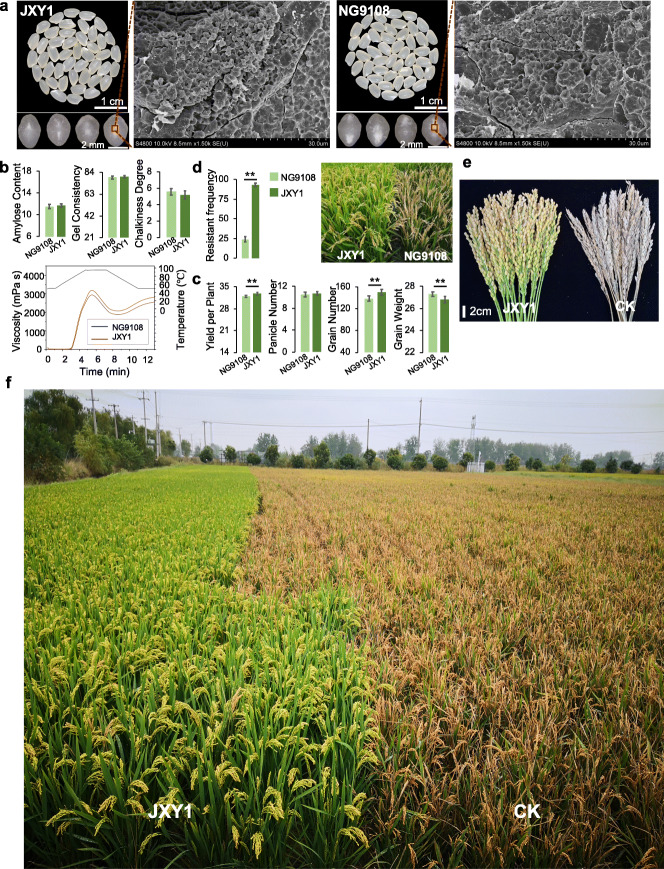
Rice blast resistance improvement in JXY1. **a** Seed phenotype and starch granule morphology of JXY1 and its recurrent parent NG9108. **b** Comparisons of taste quality between JXY1 and NG9108. **c** Comparisons of yield traits between JXY1 and NG9108; ** *P* < 0.001 by Student’s *t* test. **d** The blast resistance frequency of JXY1 carrying *Pigm* was significantly higher than that of NG9108; ** *P* < 0.001 by Student’s *t* test. **e** Compared with NG9108, JXY1 in Jiangsu natural rice blast nursery showed better resistance to rice blast, and the loss rate of a single plant was significantly lower. **f** Under natural planting conditions that did not prevent rice blast in the Jianhu region in Jiangsu province, JXY1 showed better resistance to rice blast than the susceptible varieties “Huruan1212” widely planted in this region

The second improved strategy was the precise design of new blast-resistant lines with high yield and excellent taste quality. NG9108 is the main variety cultivated widely in *japonica* rice-growing areas of central China, but its high susceptibility to rice blast poses a high risk to rice production. Hence, we used NG9108 as the recurrent parent and YG7311 carrying the blast resistance gene *Pigm* as the donor parent to develop a line with enhanced rice blast resistance with a high yield and excellent quality background (Fig. 7a). Using the similar process as above, we generated one elite line (JXY1) carrying both *Pigm* and *Wx*^*mp*^ and retained the superior genes for grain weight and grain number from the recurrent parent NG9108 and donor parent YG7311. In terms of quality, JXY1 and NG9108 showed no significant differences in starch grain morphology (Fig. 7a) and rice physicochemical properties (Fig. 7b). However, the yield of JXY1 was significantly higher than that of the recurrent parent NG9108, because it had acquired the grain number-related superior gene *NOG1* from YG7311 (Fig. 7c). In addition, the blast resistance of JXY1 (92.4%) was significantly enhanced compared to NG9108 (24.5%) (Fig. 7d). The yield of JXY1 was not obviously altered under the conditions of uncontrolled rice blast in the field, but the yield loss of the susceptible control was more than 90% (Fig. 7e, f).

## Discussion

Illustrating the law of genetic improvement of crops in recent years at the genomic level will not only provide insights into how important agronomic traits were improved, but also is useful to optimize the molecular design of breeding to effectively break the bottleneck effect of traditional breeding. In 2007, the concept of super green rice was highlighted, which should maintain high yield, good quality, disease resistance, insect resistance, abiotic stresses tolerance, and nutrient efficiency [41]. Among these traits, yield, quality, and disease resistance are key determinants [42]. Till 2018, 189 genes related to yield, 63 genes related to grain quality, and 221 genes related to disease resistance have been characterized [5]. However, it is still hard to merge high yield, good quality, and immunity based on these functional genomic resources because of the resource reallocation and genetic linkage drag [37]. In this study, the superior genes related to high yield, excellent quality, and blast resistance in central China have been clearly illustrated for the first time at the genome level and created a genome-wide linkage map. This useful information will not only help breeding scientists understand why varieties cannot be compatible with high yield, excellent taste, and blast resistance from a genomics perspective, but also provide ideas for designing ideal varieties with precision and high efficiency. From the present results, we know that the linkage drags on chromosome 6 are the key issue for high-yield incompatibility, excellent eating, and blast-resistant traits. During the past 30 years, the deleterious linkage-type Hap2 respected low-yield and blast-resistant is existing in most BRMVs, while the Hap3 respected high-yield and taste-excellent is existing in all BSLVs. Based on this discovery, we created a novel superior haplotype with high-yield, is blast-resistant, and is taste-excellent via breaking the deleterious linkage drags on chromosome 6.

Since the completion of whole-genome sequencing, massive genomic information has greatly promoted the development of rice genetic improvement [43]. Although more than rice 2000 genes controlling important agronomic traits have been isolated, only a few genes are used to improve specific traits in breeding practice. Recently, Wei et al. proposed a strategy of fine design breeding and realized the 57 improvement of yield and plant architecture via the RiceNavi system [18]. In this study, we identified the key genes could be used to balance yield, eating quality, and blast resistance through GWAS and complete the entire process without introducing any linkage drag, which was much quicker and more precise than conventional breeding [44–46].

Furthermore, an appropriately modified strategy was proposed in this study to enhance the precision of yield selection. We used the GS model to anchor the core recurrent parent with the best gene combination for yield, and the major superior genes (yield, taste quality and blast resistance) were strongly selected in each backcross and selfing generation. Using the above strategies, the beneficial genotypes in the breeding population can be enriched to the maximum in the shortest generation time. The selected JXY1 and XY99 lines had a higher yield potential than the lines selected by traditional breeding methods (Additional file 1: Fig. s10a). Moreover, both JXY1 and XY99 recorded high level blast resistance in natural disease nurseries in Jiangsu, Hubei, Anhui, Zhejiang, and Shandong provinces (Additional file 1: Fig. S10b-c). Therefore, genome-wide selection and enhanced selection of major genes for yield selection are efficient breeding strategies. It is foreseeable that large-scale employment of JXY1 and XY99 in the *japonica* rice-planting areas of central China can significantly reduce the quantity of chemical pesticide use, which is of exceptional significance for attaining green and safe rice production and protecting the environment at the same time.

Our study was the first large-scale genomic landscape analysis of *japonica* rice varieties grown in central China, and we demonstrated a balancing of multiple agronomic traits using a genomic-based strategy, which provided novel insights for the molecular breeding of rice and other crops.

## Conclusions

In summary, based on the large-scale genomic landscape analysis, balancing high yield, good quality, and blast resistance can be achieved by pyramiding the superior genes and breaking the linkage drag. Further, the inferior genes show strong linkage could be modified by CRISPR/Cas9 to create new varieties. And with the continuous updates of cloned genes and more sequenced rice varieties, genomic molecular design breeding based on big data will become an increasingly precise and powerful strategy for accelerating rice breeding programs in the future. This research not only provides technical and theoretical guidance for efficient and precise molecular design breeding in practice but also closes the gaps between genomic studies and crop breeding. With the development of genomics, quantitative genetics, and computational biology technology, molecular design breeding can be used more broadly.

## Methods

### Plant materials and phenotypic measurements

In total, 200 elite *japonica* rice varieties used for whole-genome sequencing were collected from the germplasm bank or breeders in central China. The selection criterion was that the varieties were planted in central China over a large-scale area. The publicly available pedigrees and registration information for all varieties are available at the Ricedata website (www.ricedata.com). The 200 varieties were planted and phenotyped across four environments: Hangzhou city in Zhejiang Province in 2017 and 2018, and Yangzhou city in Jiangsu Province in 2017 and 2018 in China. A randomized complete block design with two replicates was used in all four trials (yield, grain weight, grain number, and panicle number). Each plot contained eight rows with 10 plants per row. The row and column spacings were set to 30 and 12 cm, respectively. At least eight plants in the middle of the plot were selected for phenotyping, including panicle number per plant (PN), grain number per plant (GN), 1,000-grain weight (GW), yield per plant (YP), and amylose content (AC), according to the standard evaluation system for rice.

To obtain a best linear unbiased predictor (BLUP) value, the above trait values of four yield traits across all trials were fitted using a linear mixed model in R using the lme4 package [47] to obtain a BLUP value. Multiple comparisons of the trait values were conducted using the least significant difference (LSD) method with the R package (https://cran.r-project.org/web/packages/agricolae/).

### Evaluation of blast resistance at the seedling stage

A total of 35 *M. oryzae* isolates were collected from Jiangsu, Zhejiang, and Shandong provinces from 2016, the polymorphisms of cloned avirulence (*Avr*) genes among these isolates were analyzed, and the primers used for amplification are presented in Additional file 2: Table S8. Based on the different types of *Avr* genes in each *M. oryzae* isolate, we selected seven isolates (Suzhong7, Subei1, Subei5, Subei6, Shandong3, Sunan1, and Zhejiang3) to screen the seedling blast resistance of the 200 *japonica* varieties. Single spore isolation, strain cultivation, and inoculum preparation were conducted manually to identify resistance rice varieties. Ten plants of each *japonica* variety were grown in a plastic tray filled with sieved garden soil in a greenhouse maintained at 27–30 °C until three leaves emerged. Three-week-old rice seedlings were inoculated with 40 mL of an *M. oryzae* conidial suspension (5 × 10^4^ conidia/mL) with 0.02% Tween 20 using a hand atomizer (100 kPa) connected to an air compressor [46]. Inoculated plants were incubated for 24 h in the dark in growth chambers maintained at 26 °C. Plants were transferred to the greenhouse after inoculation under a 12 h light/12 h dark photocycle at 90% relative humidity by intermittent spraying with water. Blast disease score was recorded after 7 days according to the standard procedures [48]; rice varieties with damaged spots < 2 mm were considered resistant (R), and those with damaged spots > 1 cm were considered susceptible (S). The varieties that exhibited resistance to more than five isolates of *M. oryzae* were classified as resistant varieties, and those that showed resistance to 1–4 isolates of *M. oryzae* were classified as susceptible varieties.

### Genome sequencing and variant calling, evaluation, and annotation

Young seedlings at the three-leaf stage of the 200 *japonica* varieties were collected, and their genomic DNA was extracted using the cetyltrimethylammonium bromide (CTAB) method. The genome sequencing libraries were sequenced using an Illumina X-ten sequencer (depth range 15.0× to 30.9×). The genomic DNA of the 35 blast isolates was extracted using the Fungi Genomic DNA Extraction Kit (Solarbio, D2300). The genome sequencing libraries were sequenced using an Illumina X-ten sequencer (average depth range 30×).

The quality of the short sequencing reads was firstly evaluated using FastQC (v.0.10.1), and the reads were trimmed using Trimmomatic (v.0.36). BWA-MEM (v.0.7.13) was used to map the remaining clean reads of the *japonica* varieties, and *M. oryzae* isolates against the *Nipponbare* reference genome (IRGSP-1.0) and blast isolate (70-15) reference genomes, respectively, with default parameters. The mapping results were processed using SAMtools (v.1.3.1) [49]. GATK (HaplotypeCaller function; v.3.5-0-g36282e4) was used to call the raw variants by following the best-practice workflow. To obtain high-quality variants, we retained variants that fulfilled the following criteria: QD > 2.0, FS < 60.0, and MQ > 20.0. The low-quality variants were removed according to the following criteria: (1) missing rate > 80%, (2) frequency of heterozygous genotype > 5% or more than twice the minor homozygous allele frequency; and (3) deviation from the Hardy-Weinberg equilibrium as proposed in the GATK (excess heterozygosity < 1 × 10^−5^). Whole-genome SNPs on chromosomes and evolutionary trees were visualized using the R package CMplot (v1.0.1) and online iTOL software (https://itol.embl.de).

### Population genetics analyses

We conducted a model-based method implemented in the ADMIXTURE tool [50]. We determined the number of ancestry populations of inbred lines (K) using a ten-fold cross-validation approach implemented in the tool. When *K* = 9, the cross-validation error was sharply convergent, suggesting that *K* = 9 is a reasonable criterion for the ancestry populations of the sequenced 200 varieties. Linkage disequilibrium (LD; calculated as *r*^2^) in the study was calculated using SNPs with MAF > 0.05 and missing rate < 0.5, using Tassel (v.5.2.64) [51]. The genome-wide average *r*^2^ between two SNPs within 200-kb windows was calculated, and the distance of LD decay was represented as the physical distance over which *r*^2^ dropped to 0.2.

### Genome-wide association study of yield and blast resistance traits

We selected 2,410,743 SNPs (MAF > 0.05, missing rate < 50%) to perform GWAS for all traits. GWAS was conducted using the mixed linear model (MLM) method, which was implemented in Tassel (v.5.2.64) [51]. We performed a GWAS using both the BLUP and single-trial values for all traits. For adjacent GWAS loci (< 500 kb), loci independence was determined by pairwise linkage analysis of significant SNPs (if *r*^2^ < 0.5, they were considered independent). The confidence intervals of the GWAS loci were determined by local LD block analysis, where the pairwise *r*^2^ of the SNPs with *P* < 1 × 10^−4^ should be > 0.3. Genes located directly in or within 50 kb (genome-wide average distance of LD decay to *r*^2^ = 0.2) around the confidence intervals were selected as candidate genes for the GWAS loci. The GWAS threshold *P* value was determined using the Bonferroni correction method: threshold *P* value = 0.01/*n*, where *n* is the number of SNPs used in the GWAS analysis.

### Selective sweep detection

To detect the potential selection signals during rice breeding, we used the cross-population composite likelihood ratio (XP-CLR) [52], to scan for genome-wide selective sweeps, with a window size of 50 kb and a step size of 5 kb. The windows with the top 5% of XP-CLR scores were considered as candidate sweeps. Overlapping and adjacent selected windows were merged into a single region to represent the effect of a single selective sweep [53]. All annotated genes located directly in the sweeps were considered as potential candidate genes. Nucleotide diversity (π) was quantified using Vcftools [54] (V.4.2, http://vcftools.sourceforge.net), with a window size of 50 kb and a step of 5 kb for all chromosomes and 1 kb non-overlapping windows for candidate genes.

### Cultivated elite lines

#### Genomic prediction

The 2,410,743 SNPs and the phenotype data of the 200 sequenced varieties were used to construct a genomic prediction model using the R package rrBLUP [55, 56]. For YG7313 as the recurrent parent in the backcross generation of BC_1_F_1_ and BC_2_F_1_, two BC_2_F_1_ plants carrying all superior alleles were identified and self-crossed to obtain 242 BC_2_F_2_ individuals. Finally, 36 BC_2_F_3_ individuals were obtained, which carried the above favorable alleles. To use NG9108 as the recurrent parent, we used the same strategy, and the superior alleles detected during each backcross and selfing generation. In a later generation (BC_2_F_5_), ten recombination lines were selected to sequence the genome with a 5× resequencing depth, and the identified SNPs were used for genome-wide selection based on the constructed prediction model. The lines with the best prediction values were selected to further calculate the yield, blast resistance, and taste quality. The KASP marker information identified the genotype of grain weight (*BG3*, *OsPL3*, *OsTB1*, *TGW6*, *OsSNB*, and *OsPL18*), grain number (*NOG1* and *DEP1*), panicle number (*OsIAGLU*), taste quality (*Wx*^*mp*^), and blast resistance (*Pigm*) are shown in Additional file 2: Table S9.

### Rice quality analysis

#### Specimen preparation for scanning electron microscopy

The dried grains were broken in the middle section of the kernel with tweezers. Fractured grains were fixed on the specimen mount with the fractured surface facing upward and sputter-coated with gold before being viewed using a Hitachi S4800 scanning electron microscope at 10 kV.

#### Isolation of starch and amylose content

The seeds were ground thoroughly in a mortar and mixed with water to form a homogenate. The homogenate was filtered using 100- and 400-mesh sieves. After centrifugation (5000×*g* for 5 min), the yellow impurities on the precipitate’s surface were removed. The precipitate was then washed three times with water and twice with ethanol. The starch was oven-dried at 40 °C for 2 days and passed through a 100-mesh sieve. Dry starch (10 mg) was placed in a test tube, and the sample was immediately dissolved in 5 mL of dimethyl sulfoxide (DMSO) containing 10% 6.0 M urea. The mixing was performed at 95 °C for 1 h with intermittent vortexing to ensure full dissolution of starch. A 1.0 mL aliquot of the starch-DMSO solution and 1.0 mL of iodine solution (0.2% *I*^2^ and 2% KI, w/v) were mixed and made up to 50 mL with water and kept in the dark for 20 min. The control was prepared using the same method but without a starch sample. The amylose content was calculated from the absorbance measured at 620 nm, and the amylose content standard curve was produced using waxy corn amylopectin and potato amylose. The experiments were performed in triplicate.

#### Pasting property analysis of starch

The pasting properties of starch were determined using a rapid visco analyzer (RVA, 3D, Newport Scientific, Narrabeen, Australia). First, 3 g of milled rice flour was dispersed in 25 mL of water. The starch suspension was first kept at 50 °C for 1 min and then heated to 95 °C at a rate of 12 °C /min. The temperature was maintained at 95 °C for 2.5 min, and then the starch suspension was cooled down to 50 °C at a rate of 12 °C /min and maintained at 50 °C for 2 min.

## Supplementary Information

**Additional file 1: Fig S1** Geographical location and genetic structure of the 200 re-sequenced *japonica* rice varieties. **Fig S2** Quantile-quantile plots related to genome-wide association studies of yield-related traits. **Fig S3** Identification of candidate genes for yield-related traits. **Fig S4** Gene combinations contribute to yield level. **Fig S5** Artificial selection trend of GWAS mapped genes. **Fig S6** Genetic diversity analysis and selection of representative isolates of blast bacterium. **Fig S7** The haplotypes of avirulence genes in 36 sequenced isolates. **Fig S8** Yield decrease caused by Hap2 and artificial selection signals. **Fig S9** BSLVs show similarity of genomic structure on chromosome 6. **Fig S10** JXY1 and XY99 showed high-yield and broad-spectrum blast resistance.

**Additional file 2: Table S1**: The agronomic traits of 200 sequenced *japonica* rice varieties in four environments. **Table S2**: The descriptive statistics of yield-related traits in four environments. **Table S3**: Results of ANOVA analyses for yield-related traits. **Table S4**: GWAS mapped results relate to grain weight, grain number and panicle number. **Table S5**: Selection regions between *japonica* rice varieties before and after 2010. **Table S6**: Summary of cloned genes related to the yield, disease resistance, taste quality and heading day traits detected in this study. **Table S7**: Pasting properties of milled rice flour. **Table S8**: PCR amplication primer sequences for Avr genes. **Table S9**: KASP primer sequences for identification of the GWAS mapped genes relate to grain weight, grain number, panicle number and eating quality.

**Additional file 3.** Review history.

## Data Availability

All genome sequence data of 200 varieties are available under NCBI with the BioProject ID: PRJNA756648 [57].
