# Supplementary material for: Genomic insight into balancing high yield, good quality, and blast resistance of japonica rice
Source: Genome Biol. 2021 Oct 6;22:283. doi: 10.1186/s13059-021-02488-8 (PMC8493723; doi:10.1186/s13059-021-02488-8)
Supplement: Supplementary file 1 — Additional file 1: Fig S1 Geographical location and genetic structure of the 200 re-sequenced japonica rice varieties. Fig S2 Quantile-quantile plots related to genome-wide association studies of yield-related traits. Fig S3 Identification of candidate genes for yield-related traits. Fig S4 Gene combinations contribute to yield level. Fig S5 Artificial selection trend of GWAS mapped genes. Fig S6 Genetic diversity analysis and selection of representative isolates of blast bacterium. Fig S7 The haplotypes of avirulence genes in 36 sequenced isolates. Fig S8 Yield decrease caused by Hap2 and artificial selection signals. Fig S9 BSLVs show similarity of genomic structure on chromosome 6. Fig S10 JXY1 and XY99 showed high-yield and broad-spectrum blast resistance. [file 13059_2021_2488_MOESM1_ESM.pdf]

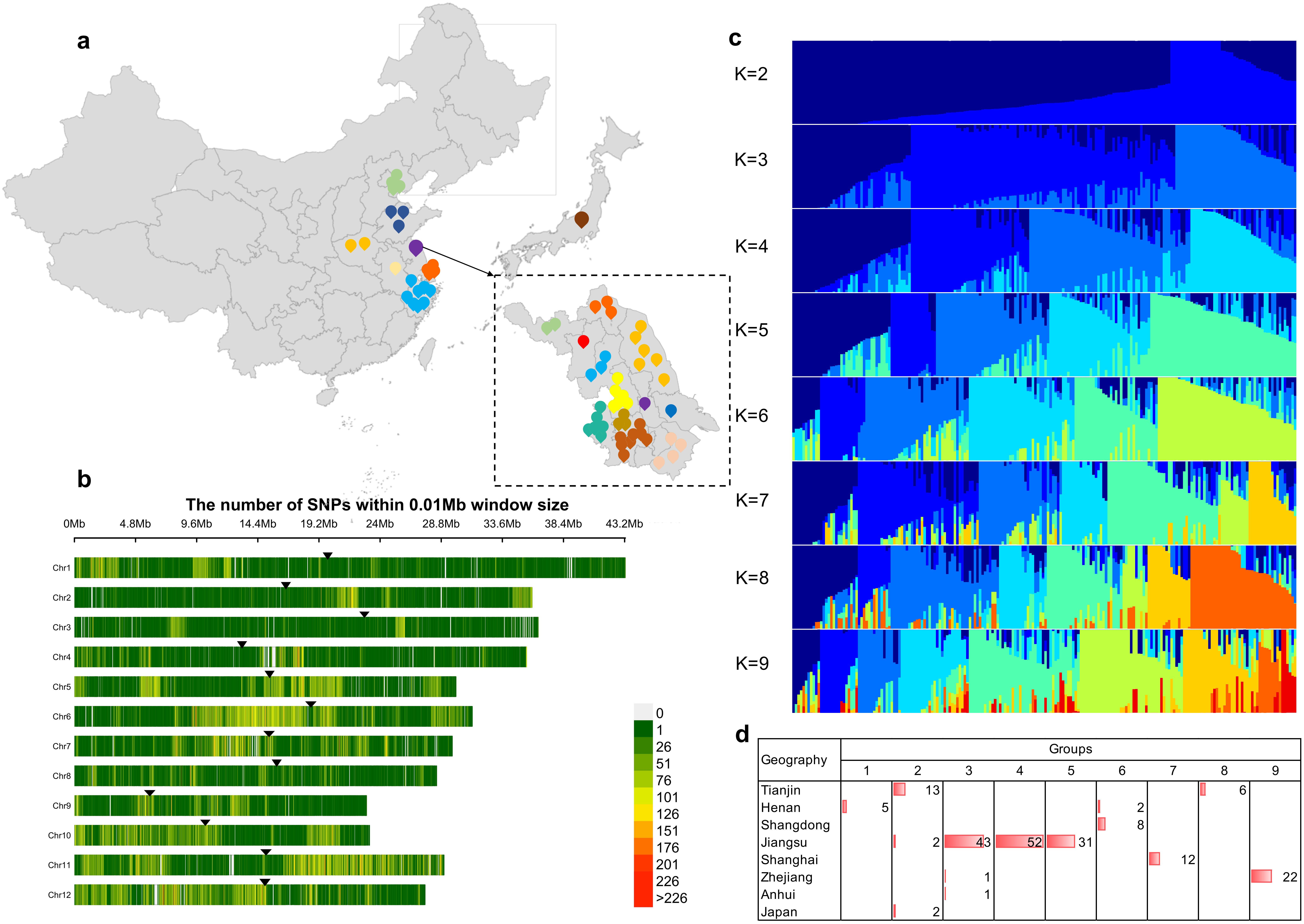

Fig. S1 Geographical location and genetic structure of the 200 re-sequenced *japonica* varieties

a Geographical location of re-sequenced *japonica* rice varieties in central China. The control variety from Japan was *Nipponbare*. The geographical location of the varieties is marked with colored dots, and a colored dot represents no more than three varieties. **b** The single nucleotide polymorphisms (SNPs) obtained by re-sequencing (15× sequencing depth) of the 200 *japonica* rice varieties uniformly covered the whole chromosomes of rice and minor allele frequency (MAF) < 0.05. Approximately 600,000 SNP markers were randomly selected for plotting. Black triangles represent the centromere location. **c** Population structure of the 200 re-sequenced varieties according to ADMIXTURE analysis. **d** Distribution of varieties from different geographical locations in each genetic group.

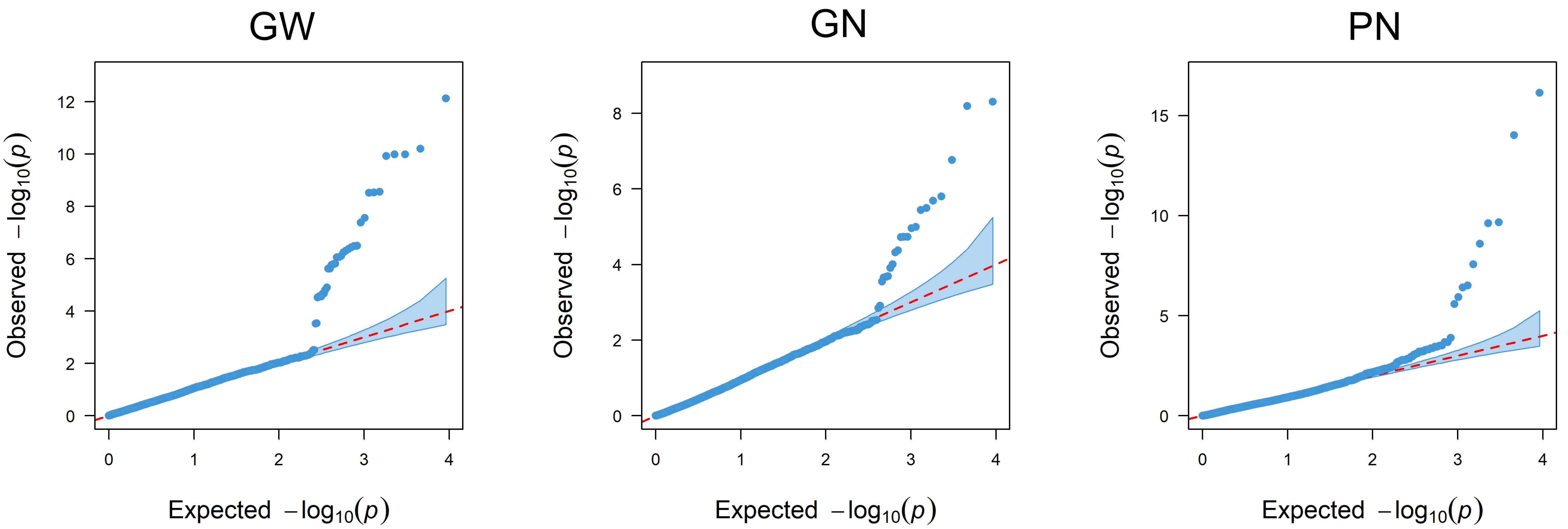

Fig. S2 Quantile-quantile plots relate to genome-wide association studies of yield related traits

GW, 1000-grain weight (unit: g); PN, panicle number per plant; GN, grain number; AC, amylose content (unit: %).

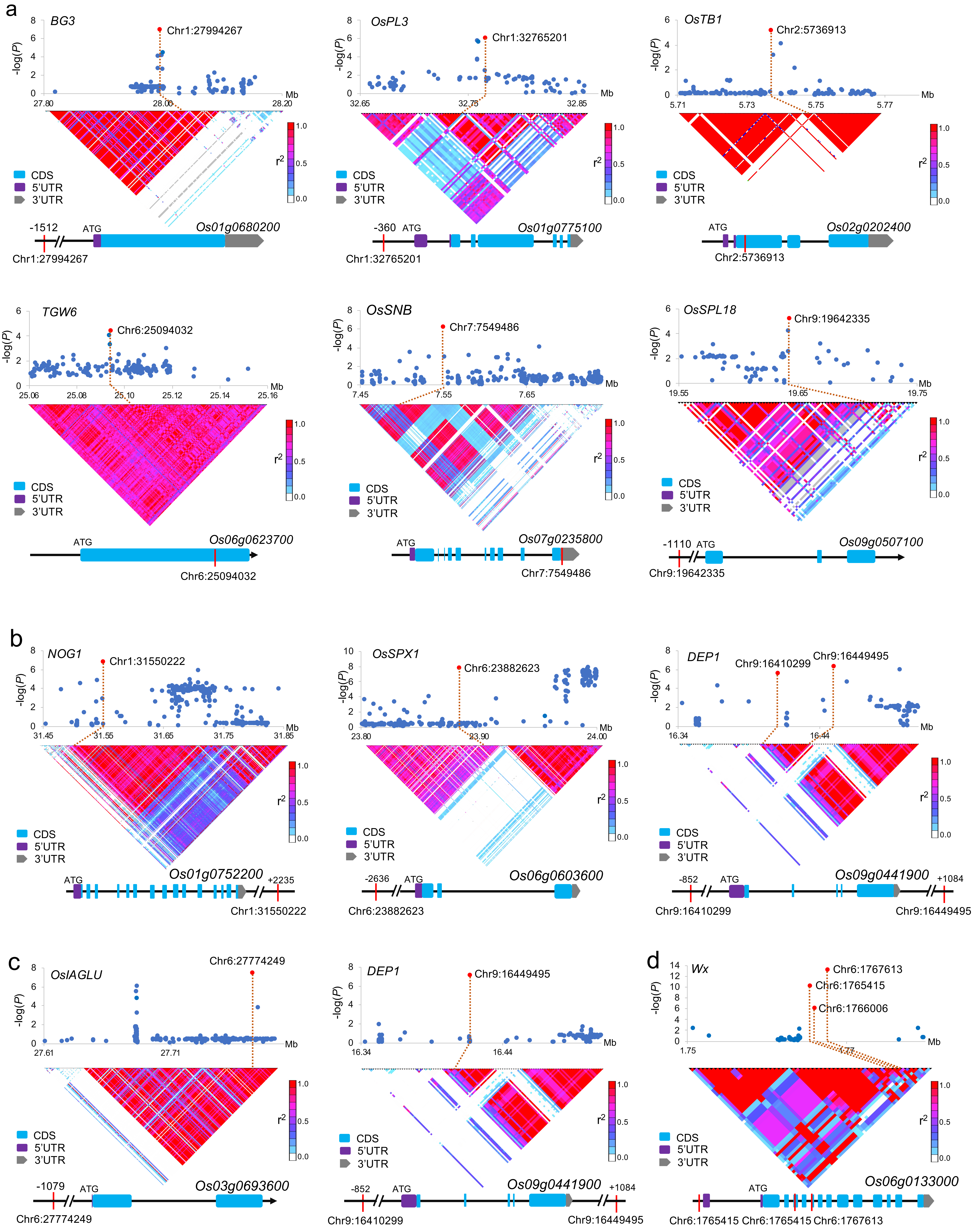

Fig. S3 Identification of candidate genes for yield related traits

a, b, c, d GWAS identification of candidate genes for GW, GN, PN, and AC, respectively. The group of plots includes a Manhattan plot (upper), an LD heat map (Middle), and candidate gene structure and putative causal polymorphisms (Lower). Peak SNP markers and putative causal polymorphisms are marked in red and their positions in the LD heat map are indicated by red lines. YP, yield per plant (g); GW, 1000-grain weight (g); PN, panicle number per plant; GN, grain number per panicle; AC, amylose content (%); GWAS, genome wide association study; LD, linkage disequilibrium; SNP, single nucleotide polymorphism.

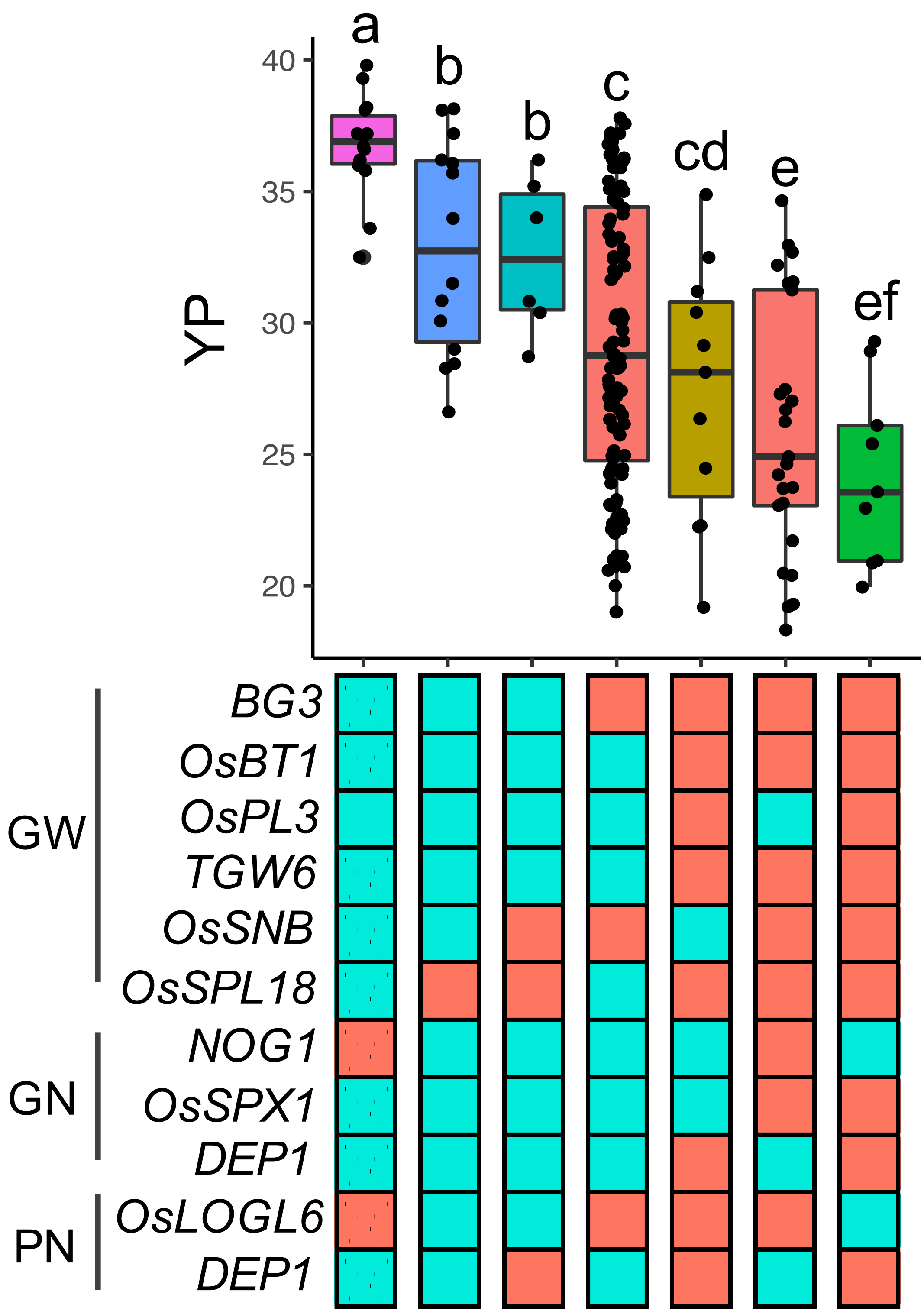

Fig. S4 Gene combinations contribute to yield level

Effects of gene combinations of grain weight (*BG3, OsBT1, OsPL3, TGW6, OsSNB*, and *OsSPL18*), grain number (*NOG1, OsSPX1*, and *DEP1*), and panicle number (*OsIAGLU* and DEP1) on yield traits; different letters indicate significant differences ($P < 0.05$ by Tukey's HSD test). Light blue represents the superior allele, and orange represents the inferior allele.

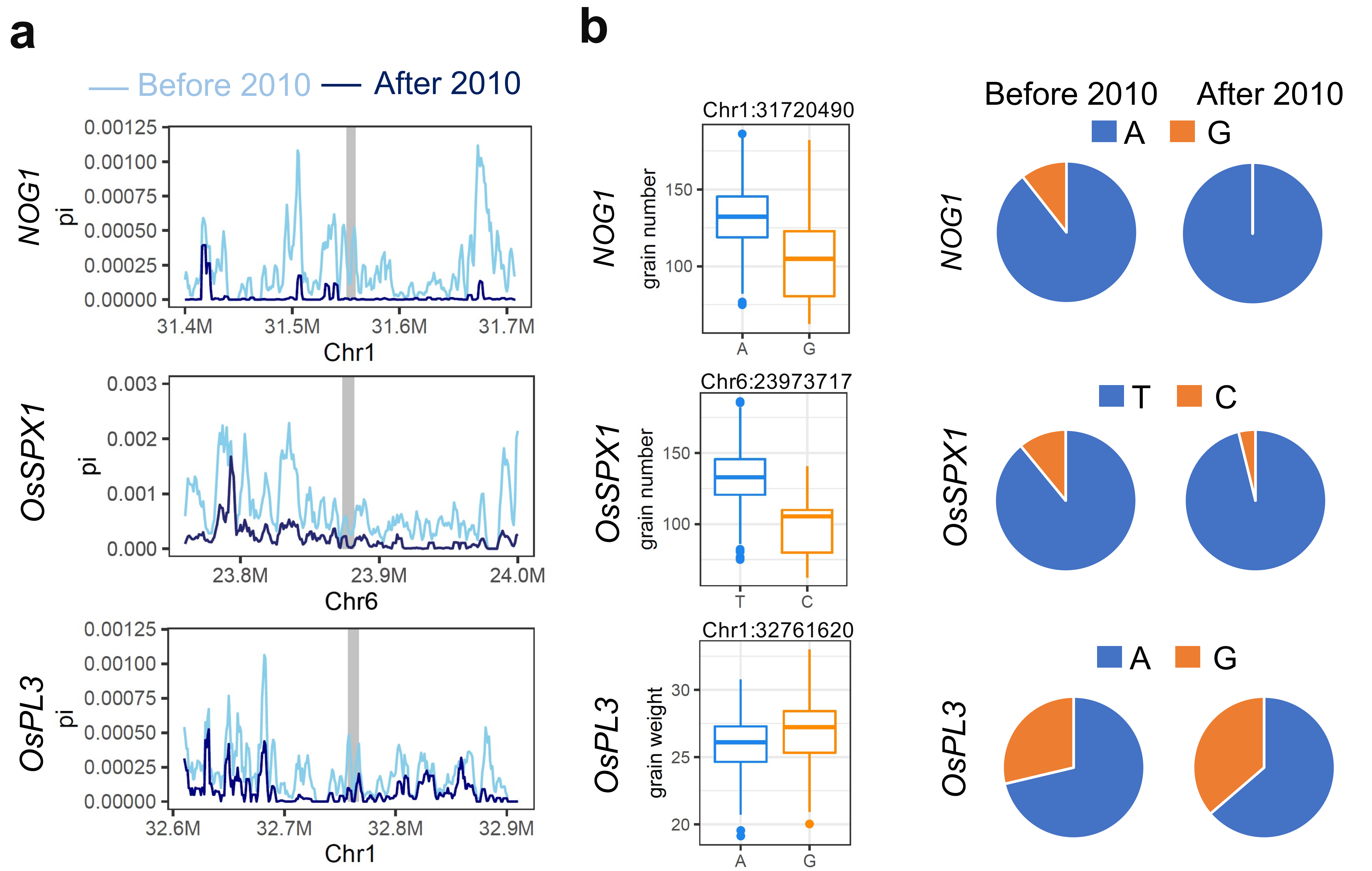

Fig. S5 Artificial selection trend of GWAS mapped genes

a Nucleotide diversity (π) near *OsPL3*, *NOG1*, and *OsSPX1* and favorable allelic genotype analysis: The pi values of chromosome regions near *NOG1*, *OsSPX1*, and *OsPL3* showed a downward trend. **b** Grain number and grain weight in varieties carrying different SNPs in *NOG1*, *OsSPX1* and *OsPL3*. Excellent allelic variants of *NOG1*^{A}, *OsSPX1*^{T}, and *OsPL3*^{G} showed an accumulation trend in the population after 2010.

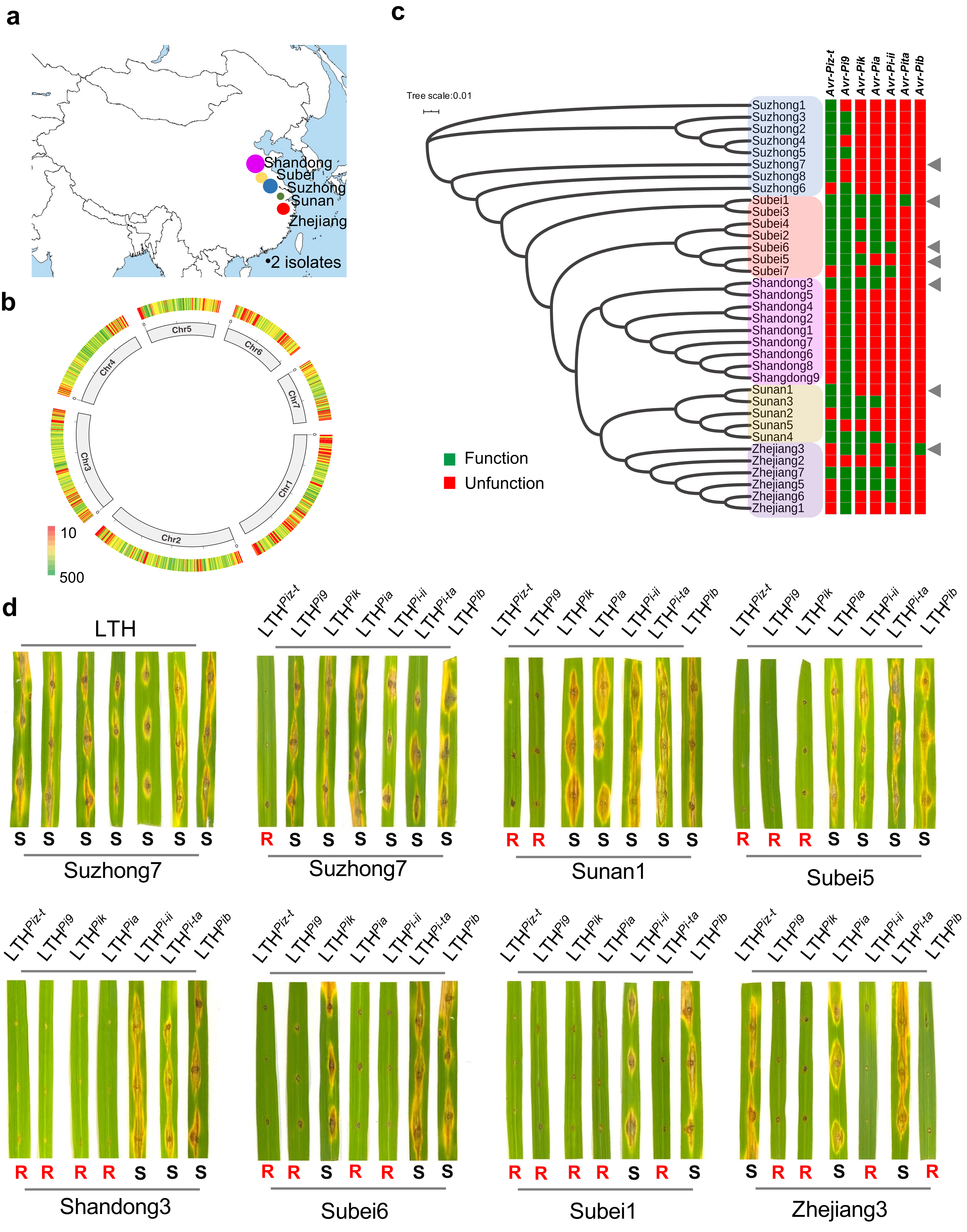

Fig. S6 Genetic diversity analysis and selection of representative isolates of blast

a Geographical location of 36 isolates of *Magnaporthe oryzae* collected; **b** A total of 85,335 SNPs obtained from 36 isolates of *M. oryzae* by sequencing at 30× sequencing depth. The reference genome was *M. oryzae* (70-15), and SNPs were filtered based on a minor allele frequency (MAF) > 0.05. **c** Genome-wide SNP markers were used to classify the genetic groups of the 36 sequenced isolates and identify the functional avirulence genes among these isolates. Green represents no mutation in the avirulence genes, red represents deletions or insertions that caused unfunctional mutations in the avirulence genes, and gray arrows represent the selected representative isolates used to assess the blast resistance of the *japonica* varieties. **d** Seedling blast phenotype of monogenic lines of *Pi2, Pi9, Piz-t, Pik, Pia, Pi-ii*, and *Pi-ta* infected by seven selected isolates. All monogenic lines had the same genetic background (Heigu of Lijiang Xintuan; LHT), R blast resistance phenotype with blast spot diameter <1 cm, S sensitive phenotype with blast spot diameter >2 cm.

a

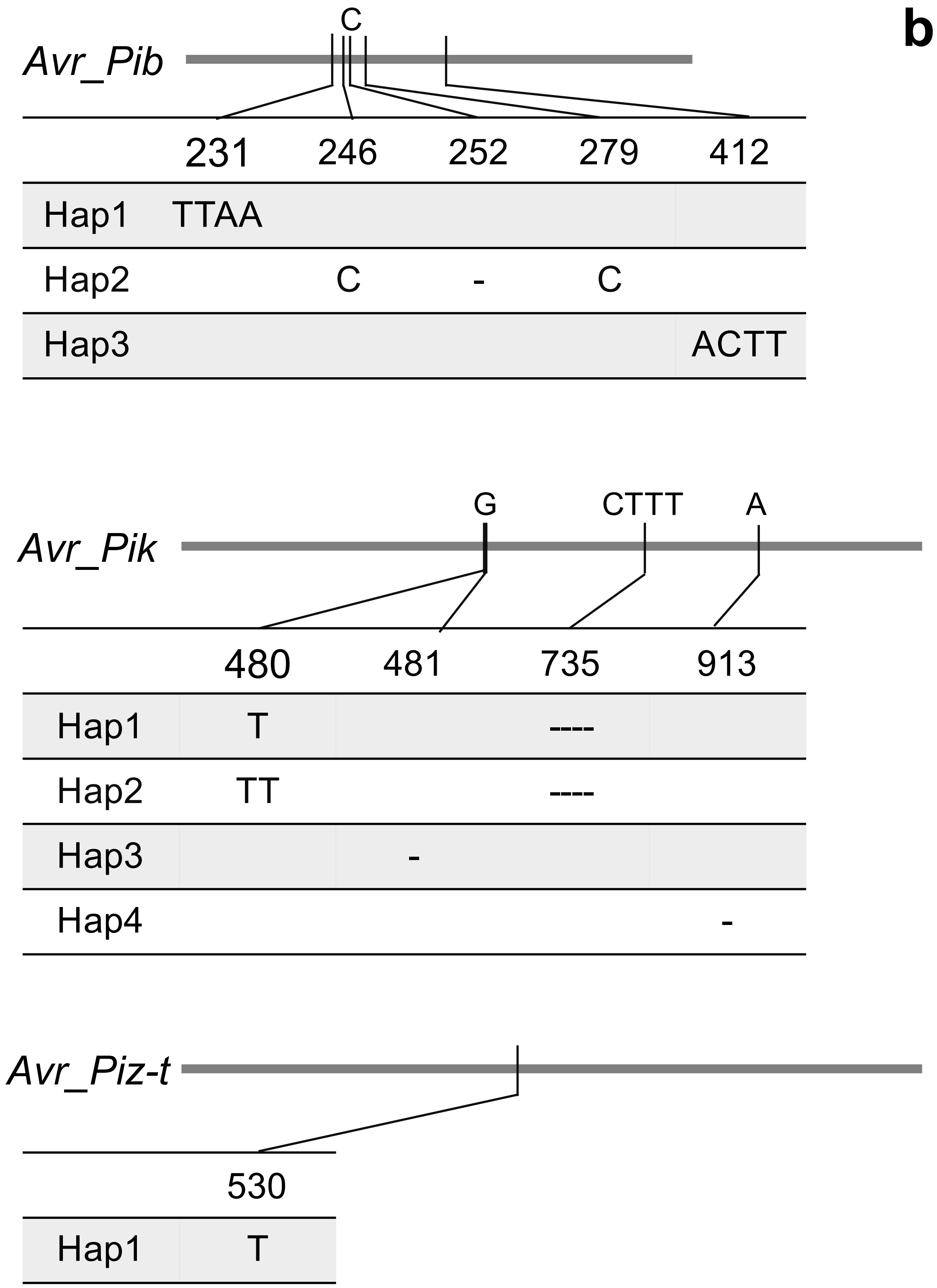

b

Isolate name	*Avr_Pi-9*	*Avr_Pi-a*	*Avr_Pi-ii*	*Avr_Pi-z^t*	*Avr_Pi-k*	*Avr_Pi-ta*	*Avr_Pi-b*
Suzhong1				+	Hap3		hap2
Suzhong3	+			+	Hap3		hap2
Suzhong2	+			+	Hap3		Hap1
Suzhong4				+	Hap3		Hap1
Suzhong5	+			+	Hap3		hap2
Suzhong7				+	Hap3		hap2
Suzhong8				+	Hap3		hap2
Suzhong6	+			Hap1	Hap3		hap2
Subei1	+	+		+	+	+	Hap1
Subei3	+	+		+	+		Hap1
Subei4	+	+		+	Hap1		Hap1
Subei2	+	+		+	+		Hap1
Subei6	+	+	+	+	Hap1		Hap1
Subei5	+			+	+		Hap1
Subei7	+	+	+	Hap1	Hap2		Hap1
Shandong3	+	+		+	+		Hap1
Shandong5	+			Hap1	Hap2		Hap1
Shandong4	+			Hap1	Hap2		Hap1
Shandong2	+			Hap1	Hap2		Hap1
Shandong1	+			Hap1	Hap1		Hap1
Shandong7	+			Hap1	Hap2		Hap1
Shandong8	+			Hap1	Hap1		Hap1
Shandong6	+			Hap1	Hap2		Hap1
Shangdong9	+			Hap1	Hap2		Hap1
Sunan1	+			+	Hap1		Hap3
Sunan3	+	+		+	+		Hap3
Sunan4	+	+		+	+		Hap3
Sunan2	+			Hap1	+		Hap3
Sunan5				+	Hap1		Hap3
Zhejiang3	+		+	Hap1	+		+
Zhejiang2			+	Hap1	Hap4		Hap3
Zhejiang4	+		+	Hap1	Hap4		Hap3
Zhejiang5	+	+	+	Hap1	+		Hap3
Zhejiang6	+		+	Hap1	Hap1		Hap3
Zhejiang1	+			Hap1	Hap1		Hap3
Zhenjiang7	+	+		+	+		Hap3

Fig. S7 The haplotypes of avirulence genes in 36 sequenced isolates

a Identification of inserted and deleted sequences causing unfunctional mutations **b** Distribution of different haplotypes in the 36 sequenced isolates. +: The amplified sequence was consistent with cloned avirulence genes.

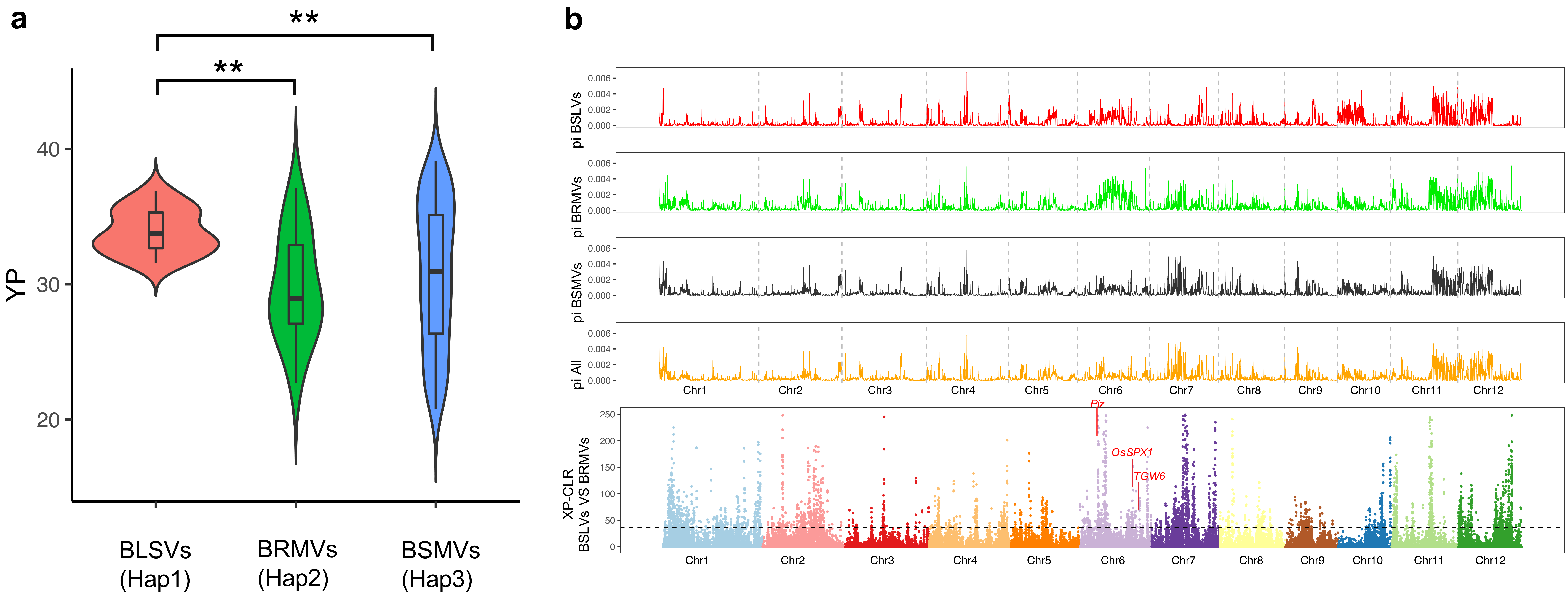

Fig. S8 Yield decrease caused by Hap2 and artificial selection signals

a Yield difference among BSLVs, BRMVs, and BSMVs, independent-samples *t*-test with P = 0.001 level, **: significant difference; **b** Genome-wide genetic diversity (π) and cross-population composite likelihood ratio (XP-CLR) between BSLVs and BRMVs. *Piz* showed strong signals for selection between BSLVs and BRMVs. BSL; blast-sensitive and low-amylose (8–12%) varieties. BRM; blast-resistant and medium-amylose (13–18%) varieties.

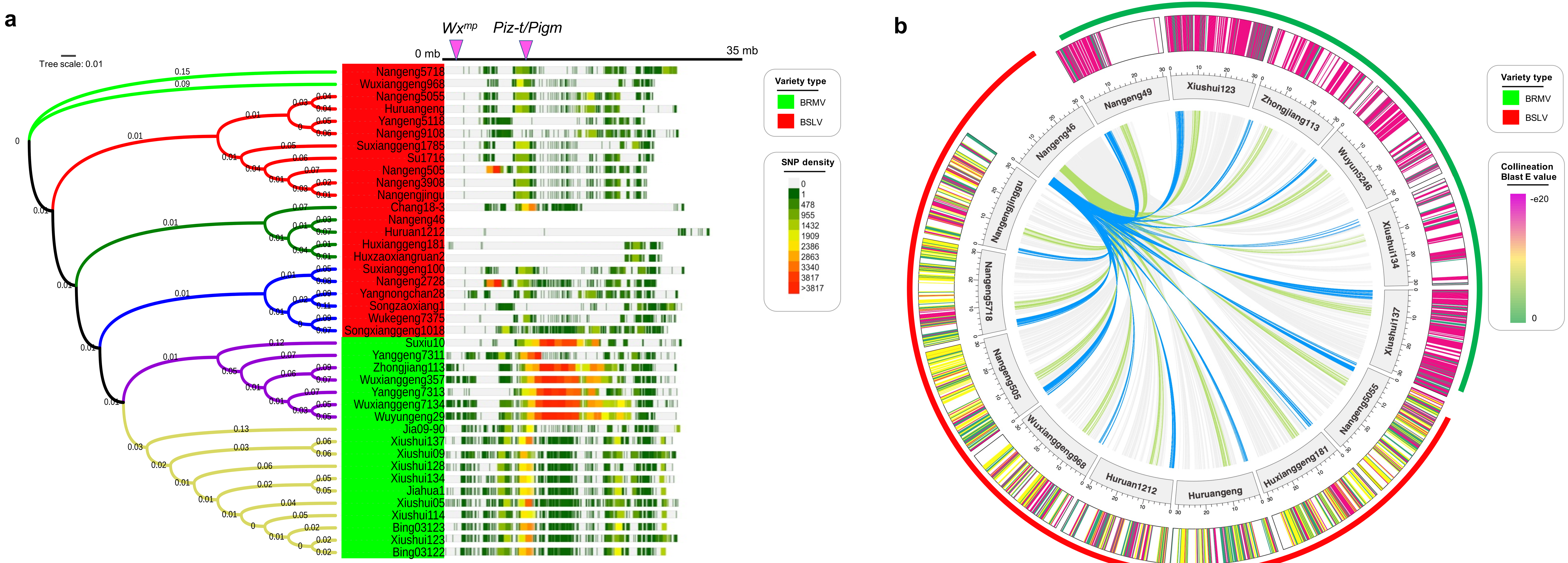

Fig. S9 BSLVs show similarity of genomic structure on chromosome 6

a Comparison of the genetic structure of chromosome 6 between BSLVs and BRMVs The blank stretches indicate that the varieties have a genetic structure similar to that of Nangeng46. Different colors represent the SNP density per 10 kb. The neighbor-joining tree lines of light green, red, dark green, and blue present the BSLVs, and purple and light yellow represent BRMVs. **b** The degree of genome collinearity of representative BSLVs (Nangengjingu, Nangeng5718, Nangeng505, Nangeng5055, Wuxianggeng968, Huruan1212, and Huxianggeng181) and BRMVs (Nangeng49/Ning74121, Xiushu123/Bing03-123, Zhongjiang113, Wuyun5246, Xiushui134/Bing06-134), and Xiushui137). The *P*-value of BLAST in each unit (200 kb) indicates the degree of similarity of chromosome fragments between each variety. BSLVs; blast-sensitive and low-amylose (8–12%) varieties. BRMVs; blast-resistant and medium-amylose (13–18%) varieties. BSMVs; blast-sensitive, and medium-amylose (13–18%) varieties. Inner blue and green lines represent the regions that contain the *Wx* and *Piz* loci, respectively. The blue lines cover the 0–4 Mb region and the green lines cover the 8–12 Mb region on chromosome 6.

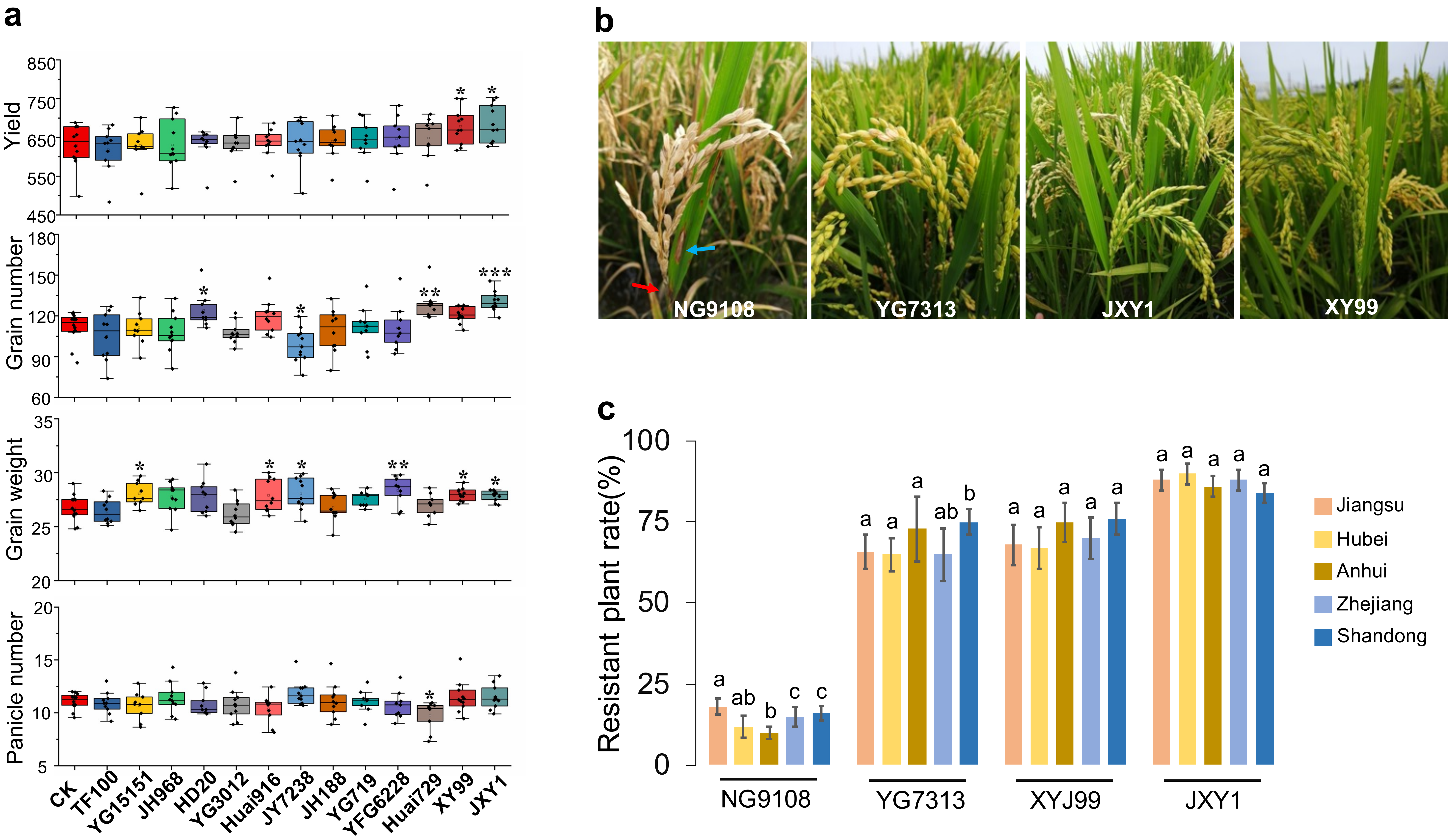

Fig. S10 JXY1 and XY99 showed high-yield and broad-spectrum blast resistance

a Comparisons of yield traits among XY99, JXY1 and other lines. * indicates P<0.05 and ** indicates P<0.01 in student's t-test between selected lines and control (Huaidao5). **b** Phenotypes of NG9108, YG7313, XY99, and JXY1 at mature stage under natural growth conditions. The red arrow represents the neck blast site, and the blue arrow signifies the typical susceptibility spot on the flag leaf. **c** Percentage of blast resistant plants in NG9108, YG7313, XY99, and JXY1 grown under natural conditions in five different provinces in Central China. Different letters indicate significant differences (P < 0.05 by Tukey's HSD test).
