# Supplementary material for: Genomic insight into balancing high yield, good quality, and blast resistance of japonica rice
Source: Genome Biol. 2021 Oct 6;22:283. doi: 10.1186/s13059-021-02488-8 (PMC8493723; doi:10.1186/s13059-021-02488-8)
Supplement: Supplementary file 3 — Additional file 3. Review history. [file 13059_2021_2488_MOESM3_ESM.docx]

Review History

**First round of review**

**Reviewer 1**

**Are you able to assess all statistics in the manuscript, including the appropriateness of statistical tests used?**

Yes, and I have assessed the statistics in my report.

**Comments to author:**

The authors re-sequenced 200 japonica rice varieties grown in central China over the past 30 years. Through genome-wide association mapping and selection scans, they found the strong selection for high-yield and eating quality associated with low amylose content may have led to the loss of resistance to the rice blast fungus Magnaporthe oryzae. Besides that, the introduced effective eating quality, and blast-resistant alleles into the background of two high-yield cultivars and bred two new varieties with excellent taste, high yield and broad-spectrum blast resistance. This manuscript will be useful to other researchers. I suggest it to be accepted after the following major revision.

1.The methods are difficult for the reader to get in. The author should describe it in detail. For example, in Line 490, please give out how the threshold value was calculated in GWAS. Please provide QQ plots, which could tell us the MLM model was appropriate or not.

2. Line498 "selected windows were merged if the distance between two windows was less than 50 kb" Why merged the windows that are not adjacent.

3 Are the phenotypic identification results of 201 varieties consistent in different environments? Please provide the trait phenotype.

4 ANOVA analysis should be performed to test variations in environment, genotype and the genotype by environment interaction were significant?

5 For XY99 and JXY1, in addition to the target traits, are there any other traits that have changed?

6 No specific variety information of the 200 materials mentioned in the article was found.

7 The reference information for the formulation of some standards mentioned in the method is not complete. For example, how to confirm the concentration of the bacterial solution.

8 How many loci did these SNPs fall in? The percentage of phenotypic variations that these SNP or loci can explain? If not 100%, how to explain this "missing heritability"?

9 There are many false positives in the GWAS results. None of the GWAS results in Fig. 1 has been verified, but the corresponding genes are located as described in the text. Please elaborate on the accuracy of the GWAS results.

10 Line 71 "growth and immunity in plant usually intertwined and" is " antagonistic".

11 Line 77 "affect" should be "affecting".

12 Line 111 "indica" should be in italic.

14 Fig. S1a	Please give out the details of geographical location of re-sequenced japonica rice varieties by dots on the graph.

15 Fig S1b Please show the centromere location of each chromosome

16 Fig S1d I am a little puzzled about Fig. S1d Do you mean when K=9, the distribution of rice varieties for each group? Please use "groups" instead of "K value".

17 Fig.1a,b Please give out P value and results of the fitting curve(Maybe the diagonals in the paper). Please supply the the unit of YP GW GN PN

18 In Fig. 1b, " * indicates a significant correlation (P < 0.001)." Is the value of P correct?

19 Fig. 1c-e Please give more information of the heatmap in each graph, why the bar value is different for each heatmap in c,d,e?

20. The abbreviation information should be marked in detail in the corresponding position (e.g.: LV, MV in Fig 2a). Please carefully check other related information. For example, please give out the full name of BSLV, LV and MV in the legend of Fig.2a.

21	Line 163-164 Was the leading SNP located at the Wx gene? Please give out LD heatmap of the candidate region.

22 Fig. 2e gene symbol should be in italic.

23 Fig. 3a; Fig. S7 Please give out the meaning of color lines of the neighbor-joining tree.

24 Fig. 3b Does GWAS only use 19 BRMVs ? How GWAS could be conducted using 19 variaties?

25 Line 241 "Fig.4a" should be in bold.

26 Fig. S7b Please give out the meaning of the inner blue and green lines

27 Fig. 5b Please give out the meaning of the green and blue dots.

28 Line 466 10-5 should be 10-5.

29 Line 484 r2 should be r2. The specific abbreviations before and after should be consistent, such as LD r2 or R2?

30 There are some mistakes in the format of the references. The authors are required to check them carefully.

**Reviewer 2**

**Are you able to assess all statistics in the manuscript, including the appropriateness of statistical tests used?**

Yes, and I have assessed the statistics in my report.

**Comments to author:**

The manuscript mainly presents a GWAS result based on japonica 200 accession/varieties from central China and pay a particular attention on two newly varieties developed by authors. The authors further provided detailed genetic improvement and changes of genes controlling key agronomy traits based on their genomic sequences. The two varieties apparently contained a good combination of some alleles to balance both yield and quanlity/resistance. Overall, a good story show a process of artifical selection on a elite variety. However, the manuscript doesn't provide new findings and lack novelty. Many similar GWAS works have been done in rice. No much new GWAS loci were identified by this study. For example, the Piz resistance locus identified by this study is a known resistance locus. Meanwhile, I think there are limited contributions of the genomic data of the 200 varieties to the development of the two new vriaties, i.e. not a case of breeding by design and just provided an explaination for the two elite at genomic level.

**Authors Response**

**Point-by-point responses to the reviewers’ comments:**

Reviewer #1:

The authors re-sequenced 200 japonica rice varieties grown in central China over the past 30 years. Through genome-wide association mapping and selection scans, they found the strong selection for high-yield and eating quality associated with low amylose content may have led to the loss of resistance to the rice blast fungus Magnaporthe oryzae. Besides that, the introduced effective eating quality, and blast-resistant alleles into the background of two high-yield cultivars and bred two new varieties with excellent taste, high yield and broad-spectrum blast resistance. This manuscript will be useful to other researchers. I suggest it to be accepted after the following major revision.

*Reply: Thank you for these constructive comments on our manuscript.*

1. The methods are difficult for the reader to get in. The author should describe it in detail. For example, in Line 490, please give out how the threshold value was calculated in GWAS. Please provide QQ plots, which could tell us the MLM model was appropriate or not.

*Reply: Thank you for these good suggestions.*

*We revised the methods in detail on Page 20 from line 430 to 433, as follows: “The selection criterion was that the varieties were planted in central China over a large-scale area. The publicly available pedigrees and registration information for all varieties are available at the Ricedata website (www.ricedata.com)”.*

*On Page 21 from line 443 to 447, as follows: “the above trait values of four yield traits across all trials were fitted using a linear mixed model in R using the lme4 package [47] to obtain a BLUP value. Multiple comparisons of the trait values were conducted using the least significant difference (LSD) method with the R package (https://cran.r-project.org/web/packages/agricolae/).”*

*On Page 24 from line 513 to 515, as follows: “The GWAS threshold P value was determined using the Bonferroni correction method: threshold P value = 0.01/n, where n is the number of SNPs used in the GWAS analysis”.*

*QQ plots were analyzed based on the PCA genetic structure and the genetic relationship of 200 varieties. The results were added in Fig. S2. From the QQ plot results, it can be seen that the MLM model used in this study is appropriate. The relevant descriptions appear on Page 8 from line 160 to 161.*

2. Line498 “selected windows were merged if the distance between two windows was less than 50 kb” Why merged the windows that are not adjacent.

*Reply: Thank you for pointing this out.*

*We are sorry for this mistake. Actually, we just merged overlapped and adjacent windows into one selection sweep region, and 50 kb was the window size we used, not the distance between the windows. We corrected these descriptions on Page 24 from line 520 to 522.*

3. Are the phenotypic identification results of 201 varieties consistent in different environments? Please provide the trait phenotype.

*Reply: Many thanks for this construction suggestion.*

*Actually, a total of 200 japonica varieties were planted in four environments, including Yangzhou city in 2017 and 2018, and Hangzhou city in 2017 and 2018. A randomized complete block design with two replications was used for the trials in these four environments. The yield-related traits, including yield per plant (YP), 1000-grain weight (GW), grain number per plant (GN), and panicle per plant (PN), were tested according to the standard evaluation system for rice and the new results were added to Table S1 and the relevant descriptions appear on Page 7 from line 143 to 145.*

*According to your suggestion, we have provided the descriptive statistics of yield related traits in the four environments and the new results were added to Table S2. ANOVA analysis further revealed that the rice varieties (genotypes) showed statistically significant variance for all four traits in the different environments and the new results were added to Table S3. The relevant descriptions appear on Page 7 from line 146 to 149.*

4. ANOVA analysis should be performed to test variations in environment, genotype and the genotype by environment interaction were significant?

*Reply:* *Thank you for this good suggestion. The ANOVA analyses to test the effects of block (within the environments), genotype, environment, and genotype by environment interaction (GEI) have been performed and the new results have been added to Table S3. The relevant descriptions appear on Page 7 from line 146 to 149.*

5. For XY99 and JXY1, in addition to the target traits, are there any other traits that have changed?

*Reply: Actually, to ensure the recombinant lines retained the excellent traits from their parents, we selected the elite lines, like XY99 and JXY1, with high yield, excellent taste, and blast resistance. Neither XY99 nor JXY1 had obvious changes in other traits.*

6. No specific variety information of the 200 materials mentioned in the article was found.

*Reply: Thank you for this suggestion. We have provided a list of the varieties, including variety name, the collection region, the yield, and the information of the year of cultivation, and the new results were added to Table S1.*

7. The reference information for the formulation of some standards mentioned in the method is not complete. For example, how to confirm the concentration of the bacterial solution.

*Reply: Thank you for this suggestion. We added the reference 47 for the method used to obtain the best linear unbiased predictor (BLUP) value of yield traits; reference 48 for the concentration of the bacterial solution, and reference 53 for the selective sweep.*

8. How many loci did these SNPs fall in? The percentage of phenotypic variations that these SNP or loci can explain? If not 100%, how to explain this "missing heritability"?

*Reply: Thank you for this suggestion.*

*A total of 20 significant loci related to yield traits, and two major loci related to blast resistance and amylose content, respectively, were identified in this study.*

*The percentage of phenotypic variations that these SNP can explain have been added in Table S4 and the relevant descriptions appear on Page 8 from line 160 to 162. From the GWAS result, we can see that the SNP or loci mapped in this study cannot completely explain the traits of grain weight, grain number, and panicle number, which is also termed the "missing heritability" problem.*

*The "missing heritability" refers to those genetic variants in genome wide association studies that cannot completely explain the heritability of complex traits (Girirajan et al., 2017). Missing heritability has always been an unresolved major scientific issue in GWAS research (Young et al., 2019). Several possible mechanisms for this "missing heritability" have been proposed, including: (1) the sample size of GWAS is not large enough, the contribution of rare variants to the phenotype could not be identified in GWAS research, and the epistasis and gene interactions also could not be found in GWAS (Brachi et al., 2011). (2) The theory of "whole gene inheritance model", which points out that most genes in the genome are significantly related to the target complex quantitative traits, and these phenotype-related genes together constitute a complex gene regulatory network (Boyle et al., 2017). Genes in the network that have a greater impact on phenotypes or genes directly related to target traits are called core genes, and the other type that need to be linked to the core genes to affect the target traits are called peripheral genes (Zhang et al., 2021). The peripheral genes are not easily identified in GWAS analysis.*

*The appearance of missing heritability is due to the inability to include all the effective segregation variants of genes in the GWAS analysis population. In addition, the effective segregation variation in different populations is always different, and the mapped genes or loci in different research populations have very low overlap.*

9. There are many false positives in the GWAS results. None of the GWAS results in Fig. 1 has been verified, but the corresponding genes are located as described in the text. Please elaborate on the accuracy of the GWAS results.

*Reply: Thank you for this suggestion. In this study, a total of 20 significant loci (threshold -log(P) value > 8.3) related to grain weight, grain number, and panicle number were identified. The leading SNPs in the GWAS peak were used to identify the candidate region, and the candidate genes closest to the leading SNP and located in the same linked LD intervals were selected as candidate genes. Based on the significant SNP position and the LD decay block, we know that eleven of the loci are previously reported quantitative trait nucleotides for which the corresponding functional genes have been cloned. Therefore, we determined that the accuracy of the GWAS results is about 55% in this study. The significant SNPs linked with candidate genes and LD heatmap were analyzed and the new results were added to Fig. S3. We added the relevant descriptions on Page 8 from line 161 to 168.*

10. Line 71 "growth and immunity in plant usually intertwined and" is " antagonistic".

*Reply: Thank you for this suggestion. This has been changed to “growth and immunity in plants are usually intertwined and are antagonistic” on Page 4 in line 75.*

11. Line 77 "affect" should be "affecting".

*Reply: Thank you for the correction. We revised "affect" to "affecting" on Page 4 in line 81.*

12. Line 111 "indica" should be in italic.

*Reply: Thank you for the correction. We revised "indica" to italic style on Page 6 in line 114.*

13. Fig. S1a Please give out the details of geographical location of re-sequenced japonica rice varieties by dots on the graph.

*Reply: Thank you for this good suggestion. The colored dots representing different japonica rice varieties have been marked in the revised Fig. S1a. and the relevant descriptions appear on Page 39 from line 846 to 847.*

14. Fig S1b Please show the centromere location of each chromosome.

*Reply: Thank you for this good suggestion. We added the centromere location markers into the revised Fig S1b and added the relevant description in the figure legend on Page 40 from in line 851.*

15. Fig S1d I am a little puzzled about Fig. S1d Do you mean when K=9, the distribution of rice varieties for each group? Please use "groups" instead of "K value".

*Reply: Thank you for this good suggestion. We meant to show the distribution of rice varieties for each group when K=9. We replaced “K value” with “groups” in the revised legend of Fig. S1d.*

16. Fig.1a, b Please give out P value and results of the fitting curve (Maybe the diagonals in the paper). Please supply the unit of YP GW GN PN

*Reply: Thank you for this good suggestion. We added the P value, redrew the fitting curve for the regression lines, and replaced all the diagonals in the revised Fig. 1a and Fig. 1b. The description of P value significance level has been added to the legend for Fig. 1a and Fig. 1b on Page 35 from line 743 to 744.*

*The unit of YP, GW, GN, and PN have been added to the legend of Fig. 1a on Page 35 from line 751 to 752.*

17. In Fig. 1b, " * indicates a significant correlation (P < 0.001)." Is the value of P correct?

*Reply: Thank you for the correction. We revised the annotation in the legend for Fig. 1 on the Page 35 in line 743, as follows:*

*“***” indicates a significant correlation (P < 0.001)”.*

18. Fig. 1c-e Please give more information of the heatmap in each graph, why the bar value is different for each heatmap in c, d, e?

*Reply: Thank you for this good suggestion. The heatmap in each graph (Fig. 1c-e) shows the density of SNP markers (-log(P) value > 4) in 100 kb chromosome intervals. The SNP marker number of -log(P) > 4 in different GWAS results varies; therefore, the bar value is different for each heatmap. We added the relevant descriptions in the legend for Fig. 1a on Page 35 from line 760 to 762.*

19. The abbreviation information should be marked in detail in the corresponding position (e.g.: LV, MV in Fig 2a). Please carefully check other related information. For example, please give out the full name of BSLV, LV and MV in the legend of Fig.2a.

*Reply: Thank you for this good suggestion. We checked all figures and added the abbreviation information in the corresponding position of the figures and their legends on Page 35 from line 756 to 757.*

20. Line 163-164 Was the leading SNP located at the Wx gene? Please give out LD heatmap of the candidate region.

*Reply: Thank you for this good suggestion. Actually, our analysis indicated that the leading SNP was located at the Wx gene locus. The LD heatmap of the candidate region has been added to revised Fig. S3d. The relevant descriptions appear on Page 9 line 176.*

21. Fig. 2e gene symbol should be in italic.

*Reply: Thank you this correction. All gene symbols have been revised to italic style in revised Fig. 2e.*

22. Fig. 3a; Fig. S7 Please give out the meaning of color lines of the neighbor-joining tree.

*Reply: The colored lines of the neighbor-joining tree in Fig. 3a represent the different K group based on the Structure result. We have added the tree line color description to revised Fig. 3a.*

*The color lines of the neighbor-joining tree in Fig. S7 (current Fig. S9) represent different groups to allow our readers to easily distinguish each group. We added the relevant descriptions to figure legend on Page 42 from line 910 to 912.*

23. Fig. 3b Does GWAS only use 19 BRMVs? How GWAS could be conducted using 19 varieties?

*Reply: Thank you for the correction. The GWAS result was conducted using 200 entries, we revised this description in the legend for Fig. 3b on Page 36 in line 776.*

24. Line 241 “Fig.4a” should be in bold.

*Reply: Thank you for the correction. We have revised it on Page 12 in line 254.*

25. Fig. S7b Please give out the meaning of the inner blue and green lines

*Reply: Thank you for this good suggestion. Original Fig. S7b is current Fig. S9b. We added the descriptions of the meaning of the inner blue and green lines to the legend of Fig. S9b on Page 43 from line 919 to 921.*

26. Fig. 5b Please give out the meaning of the green and blue dots.

*Reply: Thank you for this good suggestion. The green dots, blue dots, and red dots indicate the top 40, bottom 40, and other random varieties for YP, GW, GN, and PN, respectively. We added the descriptions of the meaning of the green and blue dots in the legend of Fig. 5b on Page 38 from line 808 to 809.*

27. Line 466 10-5 should be 10-5.

*Reply: Thank you for the correction. We have revised it on Page 23 in line 490.*

28. Line 484 r2 should be r2. The specific abbreviations before and after should be consistent, such as LD r2 or R2?

*Reply: Thank you for these corrections. We have revised them on Page 23 in line 500.*

29. There are some mistakes in the format of the references. The authors are required to check them carefully.

*Reply: Thank you for this correction. We have revised the references based on the requirements of Genome Biology.*

Reviewer #2:

The manuscript mainly presents a GWAS result based on japonica 200 accession/varieties from central China and pay a particular attention on two newly varieties developed by authors. The authors further provided detailed genetic improvement and changes of genes controlling key agronomy traits based on their genomic sequences. The two varieties apparently contained a good combination of some alleles to balance both yield and quanlity/resistance. Overall, a good story show a process of artifical selection on a elite variety. However, the manuscript doesn't provide new findings and lack novelty. Many similar GWAS works have been done in rice. No much new GWAS loci were identified by this study. For example, the Piz resistance locus identified by this study is a known resistance locus. Meanwhile, I think there are limited contributions of the genomic data of the 200 varieties to the development of the two new vriaties, i.e. not a case of breeding by design and just provided an explaination for the two elite at genomic level.

*Reply: The authors are truly grateful to the reviewer for the constructive suggestion. Based on your comments, we revised relevant descriptions in Discussion section on Page 17 and Page 19 and gave the response one by one as follows.*

*We agree that many GWAS works have been done in rice. The area of japonica rice grown in Central China, which is one of the main regions producing japonica rice worldwide, exceeds 2 million hectares, and in the past 30 years, the yields of several rice varieties in this region have surpassed the yield of hybrid indica rice. In this study, all the collected 200 japonica rice varieties were bred and grown over a large-scale area from 1989 to 2017 in the region. Thus, the collected 200 japonica rice varieties represent well the trends of rice breeding in Central China. ANOVA results revealed that these varieties showed statistically significant variance for all the investigated traits, indicating that the selected varieties hold diverse characteristics for GWAS analysis. The GWAS in this study mainly focused on the gene loci related to yield, good quality, and blast resistance, which are the three most important agronomic traits in rice breeding. Based on the specificity of the population material and the focus of the research target, the GWAS in this study will greatly benefit rice breeding in Central China.*

*Illustrating the law of genetic improvement of crops in recent years at the genomic level will not only provide insights into how important agronomic traits were improved, but also is useful for to optimize the molecular design of breeding to effectively break the bottleneck effect of traditional breeding. Based on GWAS data, a total of 20 significant loci related to yield traits, and two major loci related to blast resistance and amylose content, respectively, were identified in this study. Among them, eleven mapped loci were overlap with cloned QTLs, i.e. BG3, OsPL3, OsBT1, TGW6, OsSNB, and OsSPL18, other new loci related to grain weight and number of grains per panicle were also firstly identified in this study* *(Table S4). Although only a few new GWAS loci were identified in this study, most of the identified GWAS loci were key loci related to yield, good quality, or blast resistance. This indicates that the improvement of rice breeding in Central China is closely related to the application of these key genes. In addition, these genes were also confirmed to be the key loci controlling these traits. For example, the Piz resistance locus identified by this study is a known resistance locus. Many studies have demonstrated that it confers broad-spectrum disease resistance, especially in japonica rice varieties. In this study, we found that the Piz locus plays an important role in rice blast resistance improvement in Central China. Remarkably, GWAS and genome-wide scans indicated that strong selection for high-yield and eating quality associated with low amylose content might have led to the loss of the Piz locus in rice breeding. The integrated information of GWAS and genome-wide selection detection revealed the laws of genetic improvement of japonica rice in Central China, which provided critical clue for precise genome-wide design of new varieties with balanced high yield, good quality, and blast resistance.*

*First, the genomic data of the 200 varieties is the foundation to preform GWAS. Based on the re-sequenced data, a total of 2,410,743 SNP markers covering the whole genome were obtained and then GWAS was used to detect the gene loci related to yield, good quality, or blast resistance. Before this study, the genomic data of the collected 200 japonica rice varieties was lacking. This is the first large-scale genomic landscape analysis of japonica rice varieties grown in central China. Second, the genomic data of the 200 varieties was used to resolve the genomic linkage drag in those varieties. Through genome-wide scan, we found that there are some linkage drags in the present breeding population. For example, the genomic linkage drags in chromosome 6 caused bred failure for varieties with excellent taste and blast resistance. Third, the genomic data of the 200 varieties is the foundation to select the recurrent parents and donor parents in designed breeding. For example, the yield of JXY1 was significantly higher than that of the recurrent parent NG9108 because it had acquired the superior gene NOG1, which is related to grain number, from YG7311.*

*In 2017, Zeng et al. (2017) achieved the fusion of yield and high-quality traits in the background of indica rice through the aggregation of major genes. However, how to achieve a balance among yield, quality, and disease resistance remains an unresolved issue. After the synthetic analysis of the phenotypes and genotype of the 200 japonica rice varieties in Central China, we found that a key genomic linkage drag that caused excellent taste quality and high yield correlated negatively with rice blast resistance in the present breeding population and then provided a molecular design breeding example to balance multiple agronomic traits using a genomics-based strategy. These results showed that breaking linkage drags by constructing a precise genome selection model is a novel strategy to balance high-yield, excellent quality, and blast resistance.*

*Our study is the first large-scale genomic landscape analysis of japonica rice varieties grown in Central China, and we demonstrated a balance of multiple agronomic traits using a genomics-based strategy, which provides a novel insight for the molecular breeding of rice and other crops.*

**Second round of review**

**Reviewer 1**

After reviewing the manuscript again, I think it is much better in this new version. Most questions and concerns have been solved after the authors' revision. The responses the author give us are basically accepted. The text was revised according to the reviewers' suggestions except for minor mistakes such as the wrong writing of "shoed" in line 178.

**Reviewer 2**

I still don't agree with their so-called "Precise design of novel varieties with high yield, excellent taste, and blast resistance". They also did not response my comment about their two new elites by this study: "I think there are limited contributions of the genomic data of the 200 varieties to the development of the two new varieties, i.e. not a case of breeding by design and just provided an explanation for the two elite at genomic level."

Let us estimate their times for breeding and genomic analysis by this study: Based on their method, "The 200 varieties were planted and phenotyped across four environments: Hangzhou city in Zhejiang Province in 2017 and 2018, and 434 Yangzhou city in Jiangsu Province in 2017 and 2018 in China.", their genomic analysis results should not be available before 2019 for their "precise design". Based on their breeding program at Fig. 5a, at least five years are needed to finish whole breeding process based on my experience. In other hands, it is possible to develop a new variety in two years.

In conclusion or my opinion, it is just two independent cases to support their analysis results of the 200 japonica rice varieties.

**Authors Response**

**Point-by-point responses to the reviewers’ comments:**

Reviewer #1:

After reviewing the manuscript again, I think it is much better in this new version. Most questions and concerns have been solved after the authors' revision. The responses the author give us are basically accepted. The text was revised according to the reviewers' suggestions except for minor mistakes such as the wrong writing of "shoed" in line 178.

*Reply: Thank you for your positive comments. We have revised this minor mistake in this version and marked the changed word in res. Please see the revised content as following: “Further analysis showed that 22 low-amylose varieties”.*

Reviewer #2:

I still don’t agree with their so-called “Precise design of novel varieties with high yield, excellent taste, and blast resistance”. They also did not response my comment about their two new elites by this study: “I think there are limited contributions of the genomic data of the 200 varieties to the development of the two new varieties, i.e. not a case of breeding by design and just provided an explanation for the two elite at genomic level.”

Let us estimate their times for breeding and genomic analysis by this study: Based on their method, “The 200 varieties were planted and phenotyped across four environments: Hangzhou city in Zhejiang Province in 2017 and 2018, and 434 Yangzhou city in Jiangsu Province in 2017 and 2018 in China.”, their genomic analysis results should not be available before 2019 for their “precise design”. Based on their breeding program at Fig. 5a, at least five years are needed to finish whole breeding process based on my experience. In other hands, it is possible to develop a new variety in two years. In conclusion or my opinion, it is just two independent cases to support their analysis results of the 200 japonica rice varieties.

*Reply: Illustrating the law of yield, taste quality and resistance genetic improvement of crops at the genomic level will not only provide insights into how importance of the agronomic traits’ improvement, but also present useful information for breeders to carry out design breeding. For example, Wang et al. (Nat Genet. 2020, 52(6):565-571) illustrated the whole genome selection and genetic improvement laws in the modern maize breeding process, and identified a large number of selected genes regulating the density-tolerant plant type and stress resistance, providing an important guidance for the genetic improvement of density-tolerant maize varieties.*

*In this study, due to the sensitivity of japonica rice varieties to photoperiod, the range of suitable planting areas for japonica rice varieties is very narrow, and the corresponding breeding resources are also few. During the preliminary material collection period, we have tried our best to collect 200 representative Japonica rice varieties in central China. Based on the re-sequencing data, we found a clear selected imprinting pattern that the genes related to yield, taste quality, and resistance were strongly selected among the sequenced varieties. The identified gene loci related to quality and resistance also provide important genetic resources for the improvement of the following two elite lines (JXY and XY99), and the high-density SNP marker information provided a technology support for constructing a genome-wide selection model to select excellent high-generation materials.*

*In addition, we also found that excellent tasting varieties exhibit highly susceptible to rice blast, but the genetic reason remains unclear before this study. After analyzing the whole genome-wide linkage maps among 200 sequenced varieties, we found that the genetic linkage-drag among high-yield, excellent-quality and susceptible-resistance on the chromosome 6 caused this phenomenon. Based on the above results, we developed the two elite lines with high-yield, excellent-quality and blast resistance by breaking this linkage-drag. It is no doubt that without the above genomic information based on 200 re-sequenced varieties, we could not know how to design a rational improvement program to achieve these two superior lines.*

*Based on our genome re-sequence data and precise design principle, the breeders now can pick up ideal recurrent parent and donor parent according the genome information and rice characteristics to breed excellent japonica varieties in the future. Although we performed the agronomic trait investigation in 2017 and 2018, actually we finished genome re-sequence and made around 1100 genetic crossing F1 combinations, including YG7313×NG9108 and NG9108×YG7311 since we collected all the 200 japonica rice in 2017, although we didn’t notice both combinations are excellent in that time. We agree the breeding process is time-consuming, to save time and speed up the breeding process, we performed genetic crossing and genome re-sequence assay simultaneously. Besides, japonica rice plants are sensitive to the photoperiod and the whole growth period is around three months under short-day conditions, which accelerate the rice propagation.*

*For better understanding the times for breeding and genomic analysis in this study, the breeding schedule in detail about the two elite lines and the genomic data analysis of 200 japonica to assist breeding are as follows:*

*2017, Jan. - Apr.*

*Breeding process (Paddy in Sanya, China)*

*1. Creation of about 1,100 hybrid combinations F1 using some of the 200 japonica rice.*

*Genomic data analysis*

*1. Finished genome re-sequence of the 200 japonica rice.*

*2017, May - Oct.*

*Breeding process (Paddy in Yangzhou, China)*

*1. Grew the F1 plants of the 1,100 hybrid combinations.*

*2. Agronomic trait investigation of 200 sequenced japonica.*

*Breeding process (Paddy in Hangzhou, China)*

*1. Agronomic trait investigation of 200 sequenced japonica.*

*Genomic data analysis*

*1. Analysis of the re-sequence data and acquired genotype information.*

*2017, Nov. - Dec.*

*Breeding process (Paddy in Sanya, China)*

*1. Grew the F2 populations of all combinations.*

*Genomic data analysis*

*1. Identification of the superior alleles relate to the yield, quality and blast resistance of 200 sequenced japonica.*

*2. Analysis of whole genomic linkage.*

*3. Construction of whole genome selection (GS) model for the yield, and select the high-yield parents.*

*Based on the above analysis results, we determined high-yield parents with superior allele and less linkage drags, and confirmed two elite F2 combinations for further track selection.*

*2018, Jan. - Apr.*

*Breeding process (Paddy in Sanya, China)*

*1.Developed KASP markers for theses superior alleles and utilized in breeding process.*

*2. Selected F2 plants with all superior alleles by marker assisted selection (MAS), and the selected F2 plants were backcrossed with their recurrent parents to generated the BC1F1 seeds.*

*2018, May-Aug.*

*Breeding process (Paddy in Yangzhou, China)*

*1.Planted BC1F1 lines and selected the elite plants with all superior alleles to backcross with recurrent parents and generate BC2F1 seeds. During the vegetable growth stage, the BC1F1 lines were treated with shorten light condition.*

*2.Agronomic trait investigation of 200 sequenced japonica.*

*Breeding process (Paddy in Hangzhou, China)*

*1. Agronomic trait investigation of 200 sequenced japonica.*

*2018, Sep. - Dec.*

*Breeding process (Greenhouse in Yangzhou, China).*

*1. Planted the BC2F1 lines and selected BC2F1 plants with superior alleles to acquire BC2F2 seeds.*

*Genomic data analysis*

*1. Completed yield trait investigation and corrected the yield trait data of 200 sequenced japonica.*

*2. Further check superior alleles relate to yield trait with two years data.*

*2019. Jan. - Apr.*

*Breeding process (Paddy in Sanya, China)*

*1. Planted the BC2F2 populations, and selected BC2F2 plants with superior alleles to acquire BC2F3 seeds by MAS.*

*2019. May - Aug.*

*--Breeding process (Paddy in Yangzhou, China)*

*1. Planted the BC2F3 lines, and selected BC2F3 plants with superior alleles to acquire BC2F4 seeds by MAS. During the vegetable growth stage, the BC2F3 lines were treated with shorten light condition.*

*2019, Sep. - Dec.*

*Breeding process (Greenhouse in Yangzhou, China)*

*1. Planted the BC2F4 lines, and selected BC2F4 plants with superior alleles to acquire BC2F5 seeds by MAS.*

*Genomic data analysis*

*1. Construction of whole genome selection model.*

*2020, Jan. - Apr.*

*Breeding process (Paddy in Sanya, China)*

*1. Determination of superior BC2F5 lines through GS model.*

*2020, May - Oct.*

*Breeding process*

*Conducting multi-site testing (Paddy in Jiangsu, Hubei, Anhui, Zhejiang and Shandong, China)*

*In addition, we have uploaded all the genome sequence data of 200 varieties into the NCBI SRA database with a project accession number (BioProject ID: PRJNA756648). We added the relative description in red-colored font in Method part. Right now, all varieties sequence data are available in the NCBI database. It will be beneficial for genetic researchers to further study genomic structure variation, functional genomics and whole genome selection. In order to reduce the contribution of the genomic data of the 200 varieties to the development of the two new varieties, we revised the variety to elite line and marked the changed words in red in the current version.*
